# Do proton pump inhibitors affect the effectiveness of chemotherapy in colorectal cancer patients? A systematic review with meta-analysis

**DOI:** 10.3389/fphar.2022.1048980

**Published:** 2022-12-12

**Authors:** Wan-Ying Lin, Shih-Syuan Wang, Yi-No Kang, Andrea S. Porpiglia, Yu Chang, Chin-Hsuan Huang, Ronak Bhimani, Eahab Abdul-Lattif, Muneeba Azmat, Tsu-Hsien Wang, Yu-Shiuan Lin, Yu-Cheng Chang, Kuan-Yu Chi

**Affiliations:** ^1^ Department of Family Medicine, Taipei Medical University Hospital, Taipei, Taiwan; ^2^ Department of Education, Center for Evidence-Based Medicine, Taipei Medical University Hospital, Taipei, Taiwan; ^3^ Department of Surgical Oncology, Fox Chase Cancer Center, Philadelphia, PA, United States; ^4^ Section of Neurosurgery, Department of Surgery, National Cheng Kung University Hospital, College of Medicine, National Cheng Kung University, Tainan, Taiwan; ^5^ Department of Internal Medicine, Lower Bucks Hospital, Bristol, PA, United States; ^6^ Department of Internal Medicine, Taipei Medical University Hospital, Taipei, Taiwan

**Keywords:** colorectal cancer, proton pump inhibitor (PPI), chemotherapy, systematic review, meta–analysis

## Abstract

Proton pump inhibitors (PPI), one of the most commonly prescribed medications, carry a myriad of adverse events. For colorectal cancer (CRC) patients, it still remains unclear whether the concurrent use of proton pump inhibitors (PPI) would negatively affect chemotherapy. PubMed, Medline, Embase, and Cochrane Library were searched from inception to 10 June 2022, to identify relevant studies involving CRC patients receiving chemotherapy and reporting comparative survival outcomes between PPI users and non-users. Meta-analyses were performed using random-effects models. We identified 16 studies involving 8,188 patients (PPI = 1,789; non-PPI = 6,329) receiving either capecitabine-based or fluorouracil-based regimens. The overall survival (HR, 1.02; 95% CI, 0.91 to 1.15; I^2^ = 0%) and progression-free survival (HR, 1.15; 95% CI, 0.98 to 1.35; I^2^ = 29%) were similar between PPI users and non-users in patients taking capecitabine-based regimens, with low statis-tical heterogeneity. Although the subgroup analysis indicated that early-stage cancer patients taking capecitabine monotherapy with concurrent PPI had a significantly higher disease progression rate (HR, 1.96; 95% CI, 1.21 to 3.16; I^2^ = 0%) than those who did not use PPIs, both groups had comparable all-cause mortality (HR, 1.31; 95% CI, 0.75 to 2.29; I^2^ = 0%). On the other hand, there was little difference in both OS and PFS in both early- and end-stage patients taking capecitabine combination therapy between PPI users and non-users. Conversely, the use of concomitant PPI in patients taking fluorouracil-based regimens contributed to a marginally significant higher all-cause mortality (HR, 1.18; 95% CI, 1.00 to 1.40; I^2^ = 74%), but with high statistical heterogeneity. In conclusion, PPI has little survival influence on CRC patients treated with capecitabine-based regimens, especially in patients taking capecitabine combination therapy. Thus, it should be safe for clinicians to prescribe PPI in these patients. Although patients treated with fluorouracil-based regimens with concomitant PPI trended toward higher all-cause mortality, results were subject to considerable heterogeneity.

**Systematic Review Registration:** identifier https://www.crd.york.ac.uk/prospero/display_record.php?ID=CRD42022338161

## Introduction

Proton pump inhibitor (PPI) is a ubiquitous medication among clinicians’ armamentarium and is generally, but not exclusively, prescribed for gastroesophageal reflux disease, peptic ulcer disease, prevention of NSAID-associated ulcers, and a pivotal part of H. pylori eradication (Strand et al., 2017). PPI is also the most commonly used gastric acid suppressants in cancer patients. In addition, in the National Comprehensive Cancer Network (NCCN) guidelines for antiemesis recommended the use of either histamine-2 blocker (H2-blocker) or PPI in the management of dyspepsia in cancer patients undergoing chemotherapy (Berger et al., 2017). However, long-term use of PPI carries a myriad of adverse events, including infection, chronic kidney disease, hypomagnesemia, osteoporotic fractures (Haastrup et al., 2018), and, most notably, dysbiosis effects (Routy et al., 2018), which are well-recognized for the disruption of gut microbiota with subsequent impairment of the effectiveness of immune checkpoint inhibitors and chemotherapy in cancer patients (Roy and Trinchieri, 2017). For instance, PPI has been shown to pose detrimental effects on non-small cell lung cancer patients taking either immunotherapy (Hopkins et al., 2022) or chemotherapy (Chalabi et al., 2020), and urothelial carcinoma patients taking immune checkpoint inhibitors (Hopkins et al., 2020).

However, for colorectal cancer (CRC) patients, it still remains unclear whether the concurrent use of PPI negatively affects chemotherapy as various studies provided conflicting findings. There is concern that with concomitant use of oral chemotherapy agents and PPI, the former would be less effective. Although a *post hoc* analysis (Kichenadasse et al., 2021) of the N016966 trial (Saltz et al., 2008) suggested that there was no difference in both overall survival (OS) and progression-free survival (PFS) between PPI users and non-users in patients taking capecitabine combined with oxaliplatin (CapeOx), Sun et al. (2016) reported a significant increase in disease recurrence in patients with concomitant PPI, with a 5-year recurrence free survival rate of 74% for PPI users as opposed to 83% for non-users. On the other hand, Wang et al. (2017) showed PPI possessed a positive survival influence on patients receiving FOLFOX chemotherapy, demonstrating PPI increases chemosensitivity in CRC cells arguing that an acidic microenvironment may increase chemoresistance.

A recent systematic review (Patel et al., 2021) suggested that concomitant use of PPI in patients taking capecitabine may result in poorer oncologic outcomes. However, studies included in this review contained contradictory results. For example, Kim et al. (2021) concluded that PPI had no negative impact on capecitabine-based chemotherapy while Wong et al. (2019) found the use of PPI contributed to a significantly higher risk of disease recurrence. Another systematic review (Viñal et al., 2020) attempted to address the same issues, but the regimen was limited to capecitabine, and a meta-analysis has yet to be undertaken. Although a pooled analysis investigated the effect of PPI on fluoropyrimidine chemotherapy (Kichenadasse et al., 2021), a systematic review process was not conducted, and two questions remained unanswered: 1) the effect of PPI on capecitabine-based chemotherapy; 2) the influence of PPI on fluoropyrimidine monotherapy. Due to conflicting results, with several areas of uncertainty in between clinical studies, we are motivated to conduct this systematic review and meta-analysis to delve further into PPI’s influence on the effectiveness of chemotherapy in CRC patients, which could be useful in further studies to understand CRC tumor microenvironment and tumor recurrences.

## Materials and methods

We performed this present systematic review and meta-analysis according to the Cochrane Handbook for Systematic Reviews of Interventions (Higgins et al., 2022) and the subsequent results were reported in accordance with the Preferred Reporting Items for Systematic Reviews and Meta-Analyses (PRISMA), and Meta-analysis Of Observational Studies in Epidemiology guidelines (MOOSE) (Supplementary Method S1, S2). The study was registered on PROS-PERO (CRD42022338161).

### Study selection

PubMed, Medline, Embase, and the Cochrane Library were searched, from inception up until 10 June 2022. Three investigators (S.S.W, E.A, and M.A) independently identified relevant studies, and discrepancies were addressed by reaching a consensus with the senior reviewers (Y.C.C and K.Y.C). Search details are presented inSupplementary Method S3.

### Eligibility criteria

The three predefined criteria for evidence selection were as follows: 1) Randomized controlled trials (RCTs), prospective or retrospective cohort studies; 2) studies involving adult patients aged over 18 with colorectal cancers receiving chemotherapy; 3) studies reporting at least one comparative survival outcome, either overall survival (OS) or progression-free survival (PFS) between PPI users and non-users irrespective of indications.

### Data extraction

Two investigators (T.H.W and Y.S.L) independently extracted relevant information from eligible articles, including 1) first author’s name with publication year, 2) study type, 3) country, 4) cancer stage, 5) chemotherapeutic regimen, 6) sample size, 7) history of prior chemotherapy, 8) PPI users, 9) PPI using window, 10) age, 11) Eastern Cooperative Oncology Group (ECOG) performance status, and 12) duration of follow up.

### Quality assessment

Two investigators (Y.C and R.B) independently completed a critical appraisal of included literature by using the Cochrane Risk of Bias tool 2.0 (Sterne et al., 2019) for RCTs, and the Risk Of Bias In Non-randomized Studies of Interventions (ROBINS-I) (Sterne et al., 2016) tool for non-RCTs. Any discrepancy was addressed through discussion with the third investigator (Y.N.K).

### Main outcomes and statistical analysis

The unadjusted hazard ratio (HR) for OS and PFS were extracted directly from included studies for subsequent pooled analysis. When studies did not report the HR but presented Kaplan-Meier survival curves instead, we acquired an estimated HR from the curves through a well-established method (Parmar et al., 1998) by using a calculation spreadsheet developed by Tierney and colleagues (Tierney et al., 2007). All estimated effects were presented with a 95% confidence interval (CI). Meta-analyses were conducted using RStudio with the “meta” package (Supplementary Method S4). The pooled estimate was based on random-effects with the restricted maximum likelihood (REML) (Harville, 1977) method due to inevitable between-trial variance. Heterogeneity was assessed using I-square (Higgins et al., 2003), with values of I^2^ < 25%, 25% < I^2^ <50%, and I^2^ > 50% indicating low, moderate, and high heterogeneity, respectively. Pre-specified sensitivity analyses included subgroup analyses based on cancer stage, different treatment modification in both capecitabine-based and fluorouracil-based regimens as well as history of prior chemotherapy, and exclusion of studies subject to critical risk of bias. Determination of statistical significance in these analyses followed common threshold (*p* < 0.05).

### Publication bias

For analyses with more than 10 comparisons, a funnel plot was created to qualitatively detect publication bias. We also applied Egger’s test to quantitatively assess significant small study effects, with *p* <0.05 indicating a positive Egger’s test. When publication bias was suspected according to the Egger’s test, we performed a sensitivity analysis using the trim-and-fill method to impute potentially missing studies and re-estimated the overall effect estimates (Duval and Tweedie, 2000; Peters et al., 2007).

## Results

**FIGURE 1:**
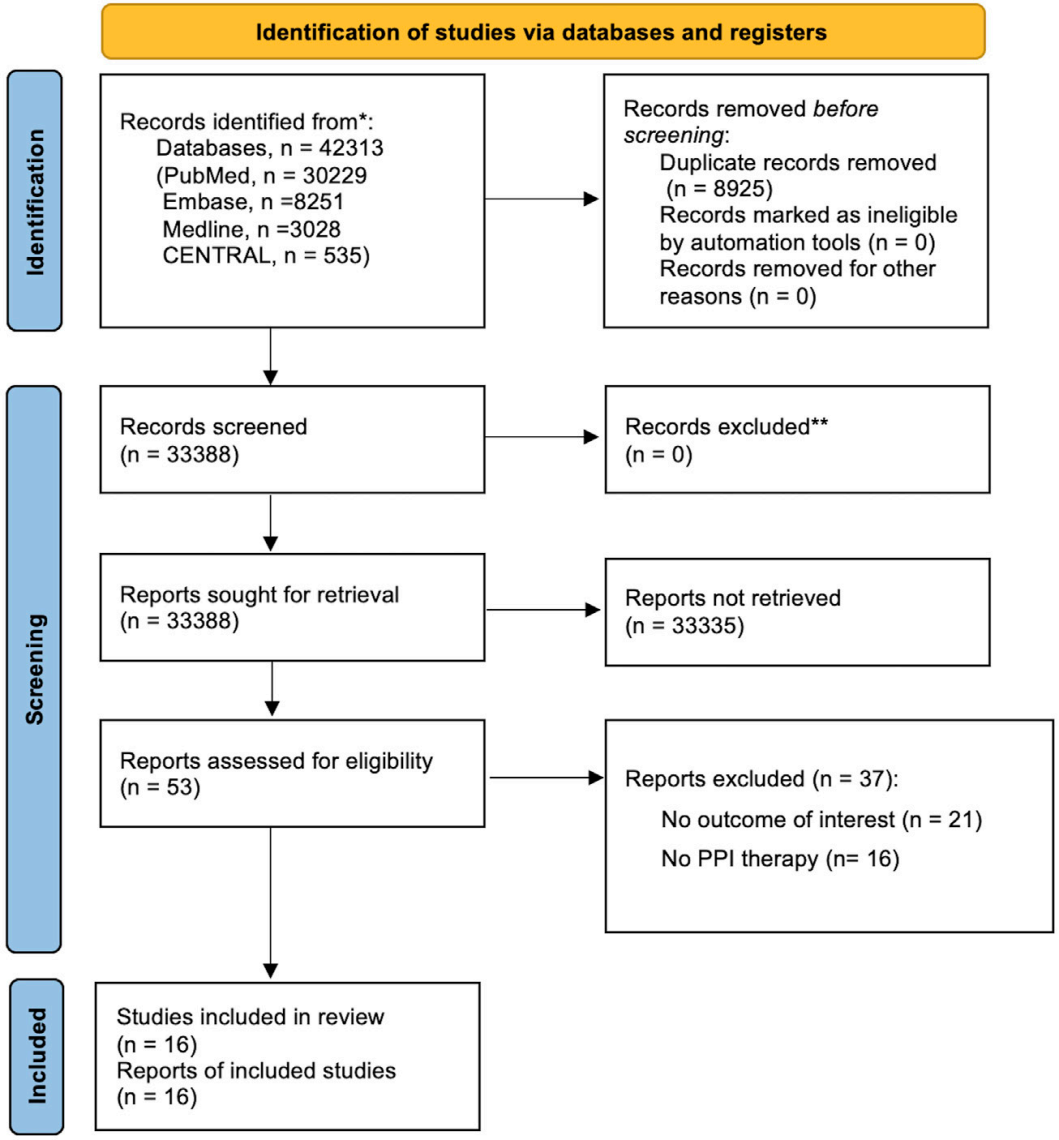
PRISMA flowchart diagram. We initially extracted a total of 42,313 potential references, including 30,229 from PubMed, 8,521 from Embase, 3,028 from Medline, and 535 from CENTRAL. After a duplicate exclusion, 33,388 studies were identified. Screening the titles and abstracts yielded 53 full-text articles, the eligibility of which was assessed. 37 studies were excluded after reading whole texts owing to reasons elaborated *in* Supplementary Results S1 in the following section. Eventually, 16 studies fulfilled the eligibility criteria and were included for qualitative and quantitative syntheses. PRISMA, Preferred Reporting Items for Systematic Reviews and Meta-Analyses.

After the systematic review, we identified 38,624 references, with 53 studies for full-text inspection, of which 37 studies did not meet the eligibility criteria (Supplementary Result S1). In the end, a total of 16 studies were included in qualitative and quantitative syntheses (Figure 1).

### Characteristics of included studies

A total of 8,118 patients (PPI = 1,789; non-PPI = 6,329) 295 enrolled between 2000 and 2022 were included in the present 296 study (Table 1). Seven are retrospective studies (Sun et al., 2016; Wang et al., 2017; Zhang et al., 2017; Rhinehart et al., 2019; Wong et al., 2019; Bridoux et al., 2022; Kitazume et al., 2022), Kim et al. (2021) is a *post hoc* analyses of AXEPT (Xu et al., 2018) trial, and Kichenadasse et al. (2021) is another *post hoc* analysis of 6 trials, including HORIZON III (Schmoll et al., 2012), N016966 (Saltz et al., 2008), Carrato 2013 (Carrato et al., 2013), VELOUR (Van Cutsem et al., 2012), RAISE (Tabernero et al., 2015), and AVF2107g (Hurwitz et al., 2004). Two *post hoc* analyses (Kichenadasse et al., 2021; Kim et al., 2021) were included for quantitative analysis, while the RCTs (Hurwitz et al., 2004; Saltz et al., 2008; Schmoll et al., 2012; Van Cutsem et al., 2012; Carrato et al., 2013; Tabernero et al., 2015; Xu et al., 2018) were included for qualitative analysis. Five studies (Sun et al., 2016; Zhang et al., 2017; Wong et al., 2019; Bridoux et al., 2022; Kitazume et al., 2022) enrolled early-stage patients, 8 studies (Hurwitz et al., 2004; Saltz et al., 2008; Schmoll et al., 2012; Van Cutsem et al., 2012; Carrato et al., 2013; Tabernero et al., 2015; Wang et al., 2017; Xu et al., 2018) investigated end-stage patients, and 1 study (Rhinehart et al., 2019) included patients of all stages. Of note, the population enrolled in AXEPT (Xu et al., 2018) trial was Asian only, and included patients from China, Japan, and Korea. Regarding the chemotherapeutic regimens, 9 studies (Saltz et al., 2008; Sun et al., 2016; Wang et al., 2017; Zhang et al., 2017; Rhinehart et al., 2019; Wong et al., 2019; Kim et al., 2021; Bridoux et al., 2022; Kitazume et al., 2022) investigated capecitabine-based regimens, of which, 3 studies (Sun et al., 2016; Rhinehart et al., 2019; Kitazume et al., 2022) examined capecitabine monotherapy, and a total of 7 studies (Saltz et al., 2008; Wang et al., 2017; Zhang et al., 2017; Xu et al., 2018; Wong et al., 2019; Bridoux et al., 2022; Kitazume et al., 2022) investigated capecitabine combination therapy, including regimens of capecitabine plus oxaliplatin (CapeOx), CapeOx plus bevacizumab (BEV), and capecitabine plus irinotecan (mXELIRI). There were another 9 studies (Hurwitz et al., 2004; Saltz et al., 2008; Schmoll et al., 2012; Van Cutsem et al., 2012; Carrato et al., 2013; Tabernero et al., 2015; Wang et al., 2017; Xu et al., 2018; Wong et al., 2019) that concentrated on fluorouracil-based regimens, with 4 studies (Saltz et al., 2008; Schmoll et al., 2012; Wang et al., 2017; Wong et al., 2019) investigating FOLFOX-based regimens, another 4 studies (Van Cutsem et al., 2012; Carrato et al., 2013; Tabernero et al., 2015; Xu et al., 2018) examining regimens of leucovorin plus continuous infusion of fluorouracil plus irinotecan, with or without ramucirumab (FOLFIRI-based regimens), and one other study (Hurwitz et al., 2004) that delved into regimens of irinotecan plus leucovorin plus bolus injection of fluorouracil, with or without BEV (IFL-based regimen). Detailed eligibility criteria for the included studies are elaborated in Supplementary Table S1. The sources of risk of bias mostly arise from bias due to confounding and the classification of interventions (Supplementary Figure S1). No study was evaluated as critical risk of bias. Supplementary Result S2 provides the detailed protocol for the ROBINS-I assessment and elaboration for each domain).

**TABLE 1:** Study characteristics.

Included studies	Study type	Country	Stage	Regimen	Sample n	History of prior chemotherapy n (%)	PPI n (%)	PPI use window	Age, mean or median (SD or IQR)	ECOG	Follow-up* (months)
*Capecitabine-based*											
Bridoux et al (2022)	Retrospective	France	I ∼ III	CapeOx	215	0 (0)	25 (11.6)	During Rx	PPI+: 61 (56–71) PPI-: 62 (54–69)	N/A	60
Kitazume et al. (2022)	Retrospective	Japan	II ∼ III	Capecitabine CapeOx	449,157	N/A	29 (6.5) 25 (15.9)	20% overlapping Rx	PPI+: 63 (58–70) PPI-: 64 (57–71) PPI+: 61 (55–66) PPI-: 60 (51–67)	N/A	60
Wong et al. (2019)	Retrospective	Canada	II ∼ III	CapeOx	214	0 (0)	50 (23.4)	During Rx	59.5 (10.1)	N/A	36
Rhinehart et al. (2019)	Retrospective	United States	I ∼ IV	Capecitabine	70	23 (32.9)	15 (21.4)	20% overlapping Rx	PPI+: 65 (26) PPI-: 73 (24)	N/A	72
Wang et al. (2017)	Retrospective	China	IV	CapeOx	364	0 (0)	215 (59.1)	During Rx	PPI+: 51.9 (N/A) PPI-: 51.2 (N/A)	N/A	60
Zhang et al. (2017)	Retrospective	China	II ∼ III	CapeOx	125	0 (0)	63 (50.4)	During Rx	55.8 (12)	N/A	60
Sun et al. (2016)	Retrospective	Canada	I ∼ III	Capecitabine	298	0 (0)	77 (26.0)	During Rx	PPI+: 68.1 (N/A) PPI-: 67.6 (N/A)	0–2	60
AXEPT (Xu et al., 2018)	RCT	China, Japan, and Korea	IV	mXELIRI	239	239 (100)	25 (10.5)	20% overlapping Rx	61 (52–67)	0–2	36
N016966 (Saltz et al., 2008)	RCT	Multiple^a^	IV	CapeOxCapeOx + BEV	637,343	0 (0) 0 (0)	96 (15.1) 52 (15.2)	During Rx	61 (18–83) 61 (18–86)	0–1	27.6
*Fluorouracil-based*											
Wong et al. (2019)	Retrospective	Canada	II ∼ III	FOLFOX	175	0 (0)	49 (28)	During Rx	59.4 (11.3)	N/A	36
Wang et al. (2017)	Retrospective	China	IV	FOLFOX	307	0 (0)	259 (70.6)	During Rx	PPI+: 51.9 (N/A) PPI-: 51.3 (N/A)	N/A	60
HORIZON III (Schmoll et al., 2012)	RCT	Europe	IV	FOLFOX + BEV	666	0 (0)	87 (13.0)	During Rx	60 (22–88)	0–1	N/A
N016966 (Saltz et al., 2008)	RCT	Multiple^a^	IV	FOLFOX FOLFOX + BEV	629,329	0 (0) 0 (0)	120 (19.1) 46 (14.0)	During Rx	60 (26–83) 60 (19–82)	0–1	27.6
AXEPT (Xu et al., 2018)	RCT	China, Japan, and Korea	IV	FOLFIRI	243	243 (100)	24 (9.9)	20% overlapping Rx	60 (51–68)	0–2	36
Carrato et al. (2013)	RCT	Multiple^b^	IV	FOLFIRI	348	0 (0)	39 (11.0)	During Rx	PPI+: 59 (52–66) PPI-: 58 (51–65)	0–1	N/A
VELOUR (Van Cutsem et al., 2012)	RCT	Multiple	IV	FOLFIRI	584	584 (100)	105 (18.0)	During Rx	PPI+: 59 (52–66) PPI-: 58 (51–65)	0–2	22.28
RAISE (Tabernero et al., 2015)	RCT	Multiple^d^	IV	FOLFIRI FOLFIRI + RAM	477,469	477 (100) 469 (100)	124 (26.0) 108 (23.0)	During Rx	PPI+: 61 (55–70) PPI-: 61 (54–68)	0–1	21.7
AVF2107g (Hurwitz et al., 2004)	RCT	Multiple^e^	IV	IFL IFL + BEV	394,386	0 (0) 0 (0)	67 (17.0) 89 (23.1)	During Rx	PPI+: 59 (52–65) PPI-: 60 (52–69)	0–1	N/A
*Post hoc analysis of RCTs*											
Kichenadasse et al. (2021) Post hoc analysis of trials of N016966, Carrato 2013, VELOUR, RAISE, and AVF2107g											
Kim et al. (2021)	Post hoc analysis of AXEPT trial										

Abbreviations: PPI, proton pump inhibitors; SD, standard deviation; IQR, interquartile range; ECOG, eastern cooperative oncology group; CapeOx, capecitabine + oxaliplatin; Rx, treatment; N/A, non-available.

RCT, randomized controlled trial; mXELIRI, capecitabine + irinotecan; BEV, bevacizumab; FOLFOX, folinic acid, fluorouracil, and oxaliplatin; FOLFIRI, leucovorin, fluorouracil, and irinotecan.

RAM, ramucirumab; IFL, irinotecan, leucovorin, and fluorouracil.

^a^ United States, South America, Canada, Europe, Australia, New Zealand, China, Hong Kong, Taiwan Korea, Thailand, Turkey, South Africa, and Israel.

^b^ Europe, Asia-Pacific, Africa, South America, and Canada.

^c^ United States, South America, Europe, Russia, Turkey, and South Africa.

^d^ United States, South America, Europe, Australia, India, Israel, Korea, Japan, and Taiwan.

^e^ United States, Australia, and New Zealand.

### Meta-analysis of capecitabine-based chemotherapy

**FIGURE 2:**
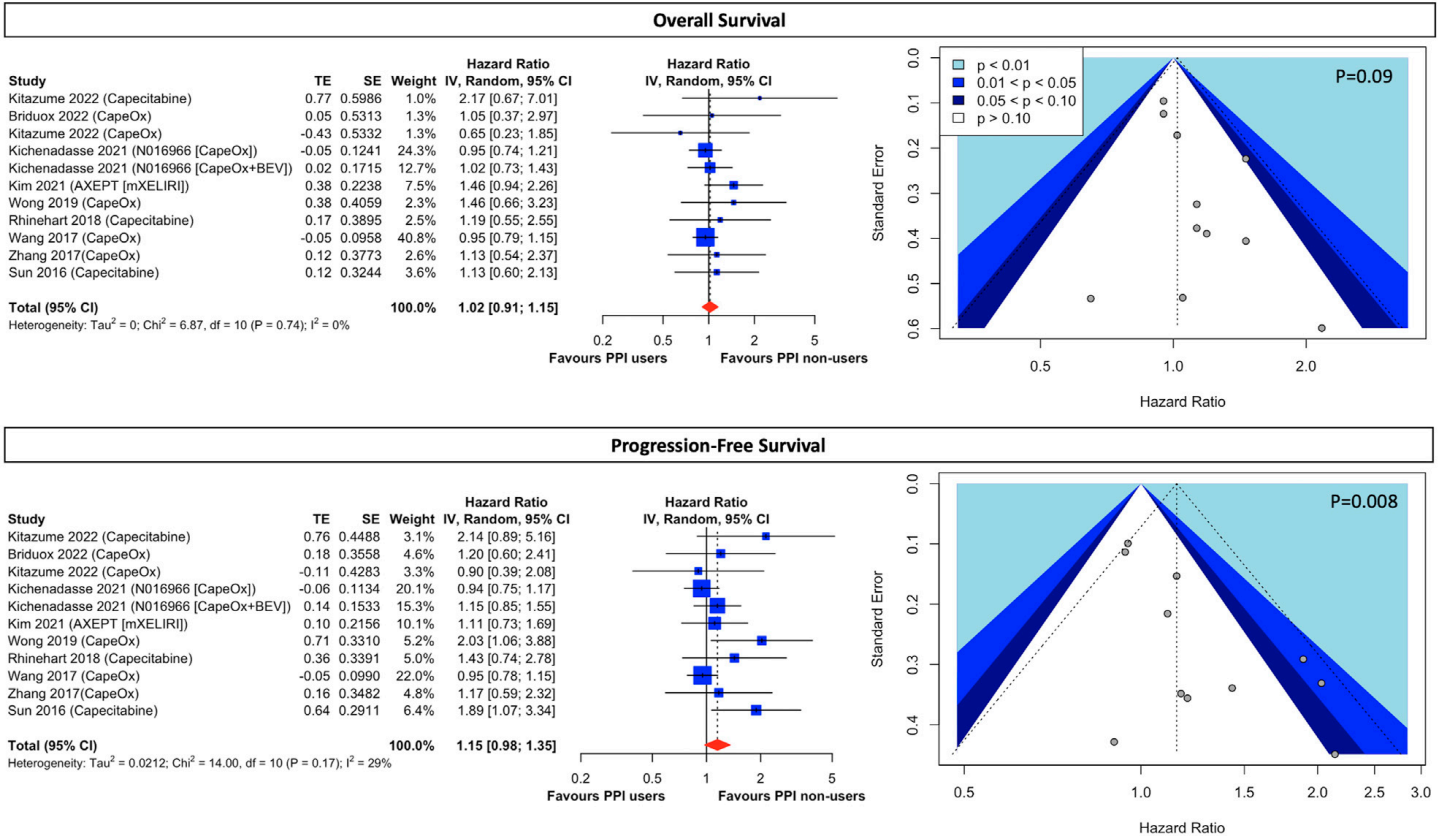
Forest plot and funnel plot of comparative overall survival and progression-free survival between PPI users and non-users in CRC cancer patients treated with capecitabine-based regimens The size of squares is proportional to the weight of each study. Horizontal lines indicate the 95% CI of each study; diamond, the pooled estimate with 95%. PPI, proton pump inhibitors; CI, confidential interval; CRC, colorectal cancers; CapeOx, capecitabine plus oxaliplatin; mXELIRI, capecitabine plus irinotecan; BEV, bevacizumab.

Our meta-analysis demonstrated that there was no significant difference in survival outcomes for both OS (HR, 1.02; 95% CI, 0.91 to 1.15; I^2^ = 0%; Figure 2) and PFS (HR, 1.15; 95% CI, 0.98 to 1.35; I^2^ = 29%; Figure 2) between concomitant PPI-users and non-users. Although scatters in the funnel appeared asymmetrical through the visualization (Figure 2), eggers’ test (Supplementary Result S3; *p* = 0.09) suggests there was no publication bias in the OS. However, regarding PFS, the funnel plot was asymmetrical and the subsequent eggers’ test detected potential small study effects (Supplementary Result S3; *p* = 0.008). Therefore, we performed a trim-and-fill analysis with 5 fictive studies being imputed, and notably, the effect estimate (HR, 1.01; 95% CI, 0.84 to 1.22; Supplementary Figure S2) did not alter significantly.

**FIGURE 3:**
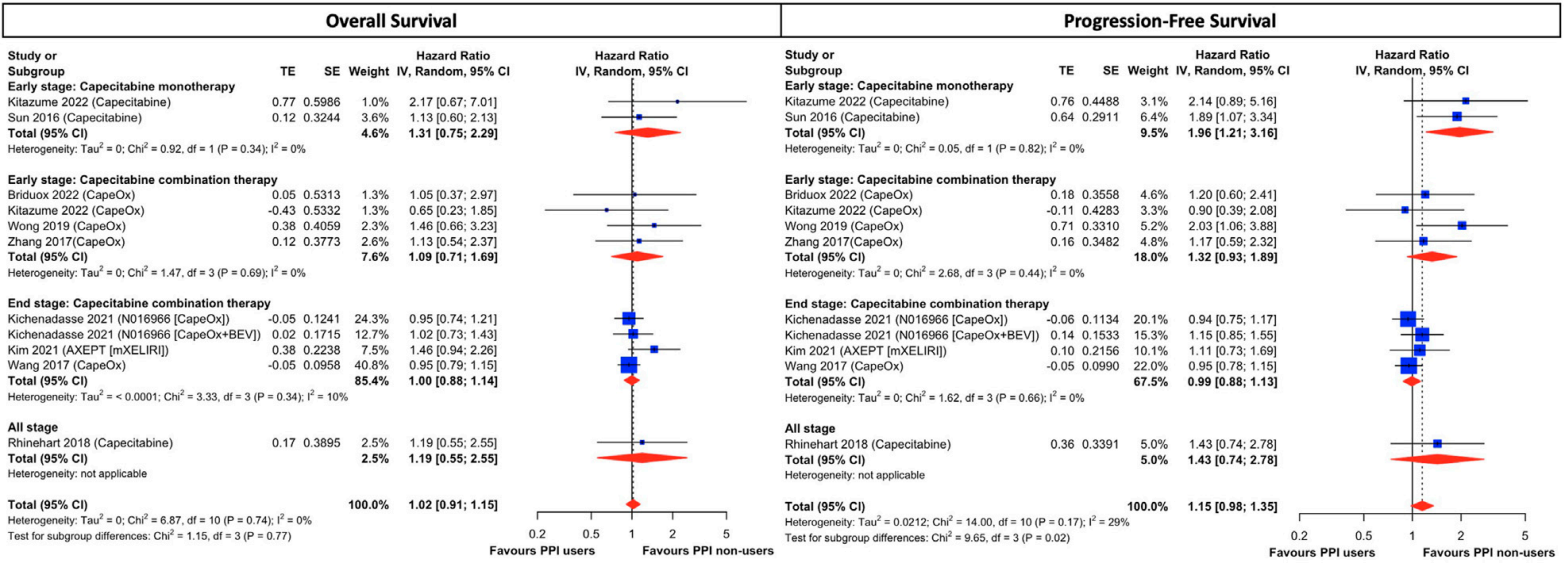
Subgroup analysis of cancer stage and treatment combination in CRC patients treated with capecitabine-based regimens. The size of squares is proportional to the weight of each study. Horizontal lines indicate the 95% CI of each study; diamond, the pooled estimate with 95%. PPI, proton pump inhibitors; CI, confidential interval; CRC, colorectal cancers; CapeOx, capecitabine plus oxaliplatin; mXELIRI, capecitabine plus irinotecan; BEV, bevacizumab.

Although results were presented with low statistical heterogeneity, conceptual heterogeneity was determined to be inevitable as cancer stages and the capecitabine-based regimens were diverse as shown in Table 1. Thus, the subgroup analysis for different cancer stages and chemotherapeutic regimens indicated that early-stage cancer patients taking capecitabine monotherapy with concurrent PPI had a significantly higher disease progression rate (HR, 1.96; 95% CI, 1.21 to 3.16; I^2^ = 0%; Figure 3) than those who did not use PPIs. However, both groups had comparable all-cause mortality (HR, 1.31; 95% CI, 0.75 to 2.29; I^2^ = 0%; Figure 3). On the other hand, there was little difference in both OS and PFS in both early- and end-stage patients taking combination therapy, including regimens of CapeOx, CapeOx plus BEV, and mXELIRI, between PPI users and non-users (Figure 3). Other prespecified sensitivity analyses (Supplementary Result S4) showed that baseline PPI use had a neutral influence on survival outcomes, irrespective of the PPI administration window (Supplementary Figure S3), and the history of prior chemotherapy (Supplementary Figure S4).

### Meta-analysis of FU-based chemotherapy

**FIGURE 4:**
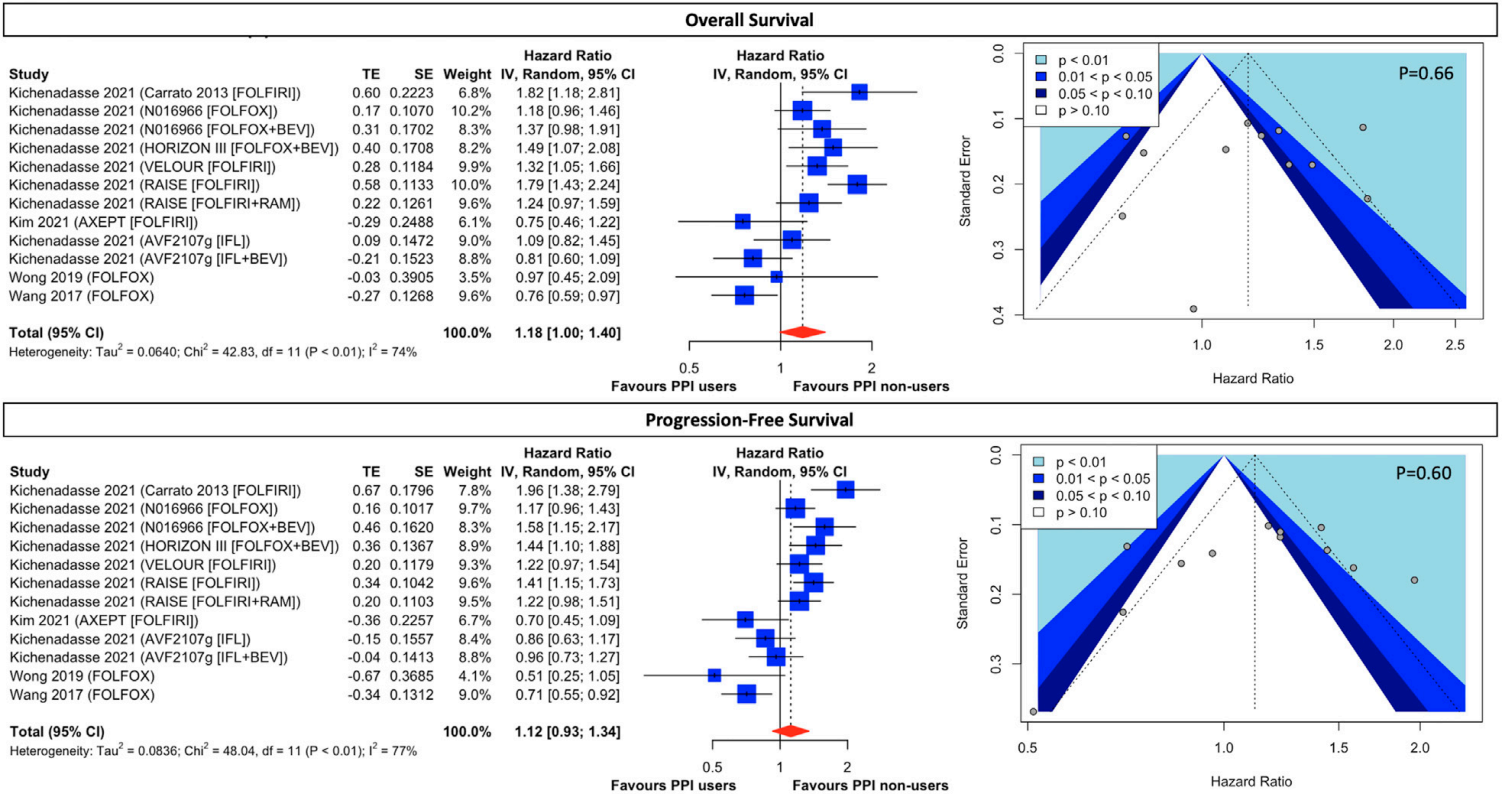
Forest plot and funnel plot of comparative overall survival and progression-free survival between PPI users and non-users in CRC cancer patients treated with fluorouracil-based regimens. The size of squares is proportional to the weight of each study. Horizontal lines indicate the 95% CI of each study; diamond, the pooled estimate with 95%. PPI, proton pump inhibitors; CI, confidential interval; CRC, colorectal cancers; FOLFOX, folinic acid, fluorouracil, and oxaliplatin; FOLFIRI, leucovorin, fluorouracil, and irinotecan; RAM, ramucirumab; IFL, irinotecan, leucovorin, and fluorouracil.

For patients receiving 5FU-based chemotherapeutic regimens, the use of concomitant PPI is associated with a marginally significant 18% higher rate of all-cause mortality (HR, 1.18; 95% CI, 1.00 to 1.40; I^2^ = 74%; Figure 4) and an insignificant 15% higher rate of disease progression (HR, 1.12; 95% CI, 0.93 to 1.34; I^2^ = 77%; Figure 4), however, with high statistical heterogeneity. Although assessment of publication bias through the visualization of the funnel plots in both OS (Figure 4) alluded to potential asymmetry, Eggers’ tests (Supplementary Result S5) are insignificant in both OS (*p* = 0.66) and PFS (*p* = 0.60). Prespecified subgroup analysis (Supplementary Result S6) of regimen modifications shows the use of baseline PPI contributes to significantly higher all-cause mortality (HR, 1.36; 95% CI, 1.04 to 1.76; I^2^ = 70%; Supplementary Figure S5) but insignificant disease progression (HR, 1.26; 95% CI, 0.96 to 1.65; I^2^ = 71%; Supplementary Figure S5) in FOLFIRI-treated patients. Albeit no survival difference between PPI users and non-users in patients treated with FOLFOX-based and IFL-based therapy, the results are still subject to considerable heterogeneity (Supplementary Figure S5). In the *post hoc* exploratory analyses (Supplementary Result S7), with the exclusion of Wong 2019 and Wang 2017 due to their retrospective nature and the exclusion of AXEPT owing to their limited enrollment of an Asian population, both FOLFIRI-treated and FOLFOX-treated patients taking PPI were associated with significantly lower OS and PFS (Supplementary Figure S6) than those without PPI, with a significant decrease in statistical heterogeneity.

### Meta-analysis using adjusted HR

Supplementary Table S3 provides variables introduced into the multivariate models for adjusted outcomes for respective studies. Moreover, Supplementary Results S8 provides the results of the meta-analysis using adjusted HR in both OS and PFS (Supplementary Figures S7–S11). It appears that there is no significant discrepancy between the unadjusted and adjusted outcomes.

## Discussion

Our study demonstrated that the survival difference was negligible between PPI users and non-users in CRC patients taking capecitabine-based chemotherapeutic regimens, regardless of cancer stages, PPI administration window and history of prior chemotherapy. The only exception was early-stage patients taking capecitabine mono-therapy with concomitant PPI, who suffered from a significantly higher risk of disease progression but comparable overall survival, compared to those without PPI. Capecitabine is an orally available prodrug that is activated through a three-step enzymatic metabolic process, initially to 5′-deoxy-5-flu- orocytidine (5′-DFCR), then to 5′-deoxy- 5-fluorouridine (5′-DFUR), and finally to 5-FU by thymidine phosphorylase in tumor tissue (Reigner et al., 2001). Our findings resonated with a recent cross-over RCT (van Doorn et al., 2022), which found that capecitabine was not negatively affected by the co-administration of esomeprazole and which surprisingly showed that the half-life of capecitabine exposure was actually pro-longed following PPI treatment. Notably, 82% patients enrolled in that RCT were CRC with ECOG <2 taking either capecitabine monotherapy or in combination with oxaliplatin and/or bevacizumab, similar to our study cohort. Moreover, Sekido et al. (2019) showed that rabeprazole did not significantly affect the pharmacokinetics of capecitabine, 5′-DFCR, 5′-DFUR, and 5-FU. Our meta-analysis in combination with these two aforementioned pharmacokinetics studies apparently contradict previous postulation (Cheng et al., 2019) that suggested increased intragastric pH levels inflicted by PPI should subsequently impair the dissolution and absorption of capecitabine, which in turn exerted detrimental effects on patients receiving capecitabine-based regimens. In fact, *in vitro* data (Röhss et al., 2004; Wishart et al., 2018) also did not support this hypothesis as the dissolution of capecitabine is similar between pH levels 2 and 6.8 with the intragastric pH level needing to reach 8.8 to become ionized before it is poorly absorbed, which is beyond PPI’s scope. Although significant asymmetry was detected in the funnel plot of PFS, it should not be interpreted abruptly as publication bias because there is a myriad of possible sources for this, including poor methodological quality, artefactual, chance, and true heterogeneity, responsible for the asymmetry (Sterne et al., 2011). In our case, the conceptual heterogeneity may be the main culprit for the observed asymmetry in the PFS since the subgroup analysis of cancer stages and regimen modifications demonstrated significant quantitative interaction between the subgroups (test for sub-group difference, *p* = 0.02; Figure 3) and the source of heterogeneity came from early-stage patients taking capecitabine monotherapy. The subgroup analysis showed the PFS remained comparable between PPI users and non-users in both early- and end-stage patients taking capecitabine combination therapy, but it was significantly lower in patients taking monotherapy with concomitant PPI. Consequently, from both a clinical and a statistical perspective, quantitative interaction between subgroups supports the idea of conceptual heterogeneity accounting for the asymmetry in the funnel plot. Even if small study effects existed to cause funnel asymmetry, based on the trim-and-fill analysis (Supplementary Figure S1), the overall effect estimates would not be altered significantly. The quantitative interaction between monotherapy and combination therapy implies to some degree that there are unknown interactions between PPI and capecitabine or PPI and CRC *per se*, which merit further investigation. Notwithstanding, although capecitabine monotherapy and combination therapy are considered to be standard postoperative adjuvant chemo-therapy for early-stage CRC (Benson et al., 2020; Benson et al., 2021), the efficacy of combination therapy has proved to be superior to monotherapy (Twelves et al., 2005; Haller et al., 2011). Thus, from a clinical perspective, it should be safe for oncologists and clinicians to prescribe PPI in patients taking capecitabine-based regimens as, theoretically, combination regimens would be prioritized and not be negatively affected by PPI.

As opposed to our study, a *post hoc* analysis of TRIO013/LOGiC trial (Hecht et al., 2016), conducted by Chu et al. (2017), indicated that concurrent use of PPI in human epidermal growth factor receptor-2 (HER-2) gastroesophageal cancer patients taking CapeOx regimen was associated with a higher all-cause mortality and a greater disease progression rate. This trial is considered a landmark study that supports the idea of detrimental effects of PPIs on capecitabine. However, two “infrequently mentioned” *post hoc* analyses of RCT, Yang et al. (2017) and Roberto et al. (2020), demonstrated PPI use during the capecitabine treatment window was not associated with decreased efficacy in advanced gastric and gastrointestinal cancers, respectively. Such variations in conclusions between Chu et al. (2017) and Yang et al. (2017) and Roberto et al. (2020) may have arisen due to the distinctive feature of the TRIO013 trial (Hecht et al., 2016), which enrolled patients with HER-2 overexpression. Studies have recognized the pivotal role of ethnicity in responses to the treatment in HER-2 amplification (Killelea et al., 2015; Yi et al., 2016) and convincing data has also suggested that the response to lapatinib, a tyrosine kinase inhibitor of HER-2, be different across ethnic groups (Hecht et al., 2016). Thus, different treatment response due to ethnicity among the cohorts may have confounded the true influence of PPI on the efficacy of capecitabine. Of note, patients in Roberto et al. (2020) were not limited to HER-2 amplification but, unfortunately, the detailed enrollment of Yang et al. (2017) remains unknown as Yang et al. (2017) was only presented as a conference abstract. Since every cancer type contains its own exclusive histopathologic profile and presents distinctive responses to chemotherapy, it is apparent that more studies are required to delve into this special population for a robust conclusion to be reached. We did not include these trials as they did not meet the eligibility criteria but we have briefly summarized the characteristics of these studies in Supplementary Table 4 as a reference for readers.

Regarding CRC patients taking FU-based regimens, those with concomitant use of PPI trend toward lower OS and PFS, compared to those without the use of PPI. However, effect estimates not only failed to reach statistical significance but were subject to considerable statistical heterogeneity, even in the pre-specified subgroup analysis of regimen modifications. We attempted to address the between-study variance by conducting *post hoc* exploratory analysis as follows. Firstly, in patients treated with FOLFOX, the most compelling source of variance is study design, with Wong et al. and Wang et al. being retrospective studies while the other 3 being RCTs. Secondly, in FOLFIRI-treated patients, although the association of PPI with significantly higher all-cause mortality suffered from residual high statistical heterogeneity, we can easily identify AXEPT (Rhinehart et al., 2019) as an outlier, alluding to it as a source of variance and qualitative interaction. Of note, the feature that distinguished AXEPT from the other three trials, Carroto et al., VELOUR, and RAISE, is the patient population. Patients enrolled in AXEPT were limited to those of Asian descent as the study was conducted in China, Korea, and Japan, contrasting with the other 3 studies which were mainly undertaken in western countries. With the removal of AXEPT from our exploratory analysis, the hazardous effects of PPI on FOLFIRI-treated patients appeared to be more consistent. We postulated that there may have been an interaction between ethnicity and treatment response, suggesting a biologic and pharmacogenetic difference between the Asian and non-Asian groups. Non-etheless, readers should bear in mind that instead of prespecified analysis, these were *post hoc* exploratory analysis, which may inevitably introduce a false interpretation of heterogeneity (Higgins et al., 2022). Thus, more studies are needed for further clarification. On the other hand, although IFL regimens share a similar combination to FOLIFRI, patients treated with IFL-based regimens experienced little survival difference between PPI users and non-users. A prospective study has already concluded that PPI did not alter the pharmacokinetics of irinotecan. Moreover, since the difference between IFL and FOLFIRI resides in the ad-ministration of fluorouracil, which is used as a bolus injection in the former and as a 48-h continuous infusion in the latter regimen, the detrimental effects of PPI on FOLFIRI instead of IFL indicate a possible interaction between fluorouracil administration and antitumor response.

The present study has various strengths. Firstly, although two systematic reviews (Viñal et al., 2020; Patel et al., 2021) attempted to address the same issues, their study design did not allow quantitative syntheses to be conducted. Secondly, two areas of uncertainty, which were proposed by Kichenadasse et al. (2021) as limitations for their study, were well-addressed: 1) the effect of PPI on capecitabine-based chemotherapy; 2) the influence of PPI on fluoropyrimidine monotherapy. Despite above mentioned novelties provided by our study, we acknowledge that our study contains several limitations. Firstly, the dose and types of PPI were not well elucidated, with this pertinent information lacking. The capability of acid suppression varied significantly in different variants of PPI (Röhss et al., 2004). In addition, it has been shown that PPI dosage exhibits a positive correlation with the magnitude and duration of gastric acid suppression (Lind et al., 2000). Secondly, the scare of information on the timing of PPI intake may hinder the assessment of association between PPI and chemotherapy, especially oral intake of capecitabine, as it takes at least 3 h for intragastric pH to attain its maximal elevation following the consumption of PPI (Hunfeld et al., 2012). Thirdly, the indications of the use of PPI were insufficient, which may have introduced unmeasured confounding bias. Last but not least, although we exhausted every chemotherapeutic regimen in current literature to explore the effect of PPI on chemotherapy, various target therapies, such as encorafenib, cetuximab, and panitumumab, used in combination with chemotherapy for stage IV CRC are still not available for investigation**.**

## Conclusion

The use of PPI has little survival influence on CRC patients treated with capecitabine combination therapy, regardless of cancer stages. Although early-stage patients taking capecitabine monotherapy with concomitant PPI were associated with significantly higher disease progression, the all-cause mortality remained comparable between PPI users and non-users. Thus, it should be safe for clinicians to prescribe PPI in these patients, especially in those patients taking capecitabine combination therapy. Conversely, both FOLFOX-treated and FOLFIRI-treated patients taking concomitant PPI trended toward higher all-cause mortality and greater disease progression, however, with considerable heterogeneity.

## Data Availability

The original contributions presented in the study are included in the article/Supplementary Material, further inquiries can be directed to the corresponding authors.
