# Supplementary material for: Do proton pump inhibitors affect the effectiveness of chemotherapy in colorectal cancer patients? A systematic review with meta-analysis
Source: Front Pharmacol. 2022 Dec 12;13:1048980. doi: 10.3389/fphar.2022.1048980 (PMC9792119; doi:10.3389/fphar.2022.1048980)
Supplement: Supplementary file 1 [file DataSheet1.DOCX]

| **Supplementary file** |
| --- |

The following material supplements the article:

*”* *Do proton pump inhibitors affect the effectiveness of chemotherapy in colorectal cancer patients? A systematic review with meta-analysis”*

**Contents of supplements**

**Supplementary Method 1.** Preferred Reporting Items for Systematic Reviews and Meta-Analyses (PRISMA) 2020 checklist

**Supplementary Method 2.** Meta-analysis Of Observational Studies in Epidemiology (MOOSE) checklist

**Supplementary Method 3.** Search strategy

**Supplementary Method 4.** Data extraction and data synthesis

**Supplementary Result 1.** Studies that were excluded following full-text inspection

**Supplementary Result 2.** Details of ROBINS-I

**Supplementary Result 3.** Assessment of publication bias in patients taking capecitabine-based chemotherapy

**Supplementary Result 4.** Prespecified sensitivity analyses of patients taking capecitabine-based chemotherapy

**Supplementary Result 5**. Assessment of publication bias in patients taking fluorouracil-based chemotherapy

**Supplementary Result 6.** Prespecified sensitivity analyses of patients taking fluorouracil-based chemotherapy

**Supplementary Result 7.** Post hoc exploratory analysis of patients taking fluorouracil-based chemotherapy

**Supplementary Result 8.** Comparative survival outcomes between PPI users and non-users using adjusted outcomes

**Supplementary Table 1.** Eligibility criteria of studies patients included in meta-analysis

**Supplementary Table 2.** Details of proton pump inhibitors

**Supplementary Table 3.** Adjusted variables of individual studies for multivariate models

**Supplementary Table 4.** Non-CRC studies assessing the association between PPI and capecitabine

*This supplementary material has been provided by the authors to give readers additional information about their work.*

**Supplementary Method 1.** PRISMA 2020 checklist

| **Section and Topic** | **Item #** | **Checklist item** | **Location where item is reported** |
| --- | --- | --- | --- |
| **TITLE** | | |  |
| Title | 1 | Identify the report as a systematic review. | Page 1 |
| **ABSTRACT** | | |  |
| Abstract | 2 | See the PRISMA 2020 for Abstracts checklist. | Page 4, 5 |
| **INTRODUCTION** | | |  |
| Rationale | 3 | Describe the rationale for the review in the context of existing knowledge. | Page 6, 7 |
| Objectives | 4 | Provide an explicit statement of the objective(s) or question(s) the review addresses. | Page 7 |
| **METHODS** | | |  |
| Eligibility criteria | 5 | Specify the inclusion and exclusion criteria for the review and how studies were grouped for the syntheses. | Page 8 |
| Information sources | 6 | Specify all databases, registers, websites, organisations, reference lists and other sources searched or consulted to identify studies. Specify the date when each source was last searched or consulted. | Page 7, 8 |
| Search strategy | 7 | Present the full search strategies for all databases, registers and websites, including any filters and limits used. | eMetohd 3 |
| Selection process | 8 | Specify the methods used to decide whether a study met the inclusion criteria of the review, including how many reviewers screened each record and each report retrieved, whether they worked independently, and if applicable, details of automation tools used in the process. | Page 7, 8 |
| Data collection process | 9 | Specify the methods used to collect data from reports, including how many reviewers collected data from each report, whether they worked independently, any processes for obtaining or confirming data from study investigators, and if applicable, details of automation tools used in the process. | Page 8 |
| Data items | 10a | List and define all outcomes for which data were sought. Specify whether all results that were compatible with each outcome domain in each study were sought (e.g. for all measures, time points, analyses), and if not, the methods used to decide which results to collect. | Page 8 |
|  | 10b | List and define all other variables for which data were sought (e.g. participant and intervention characteristics, funding sources). Describe any assumptions made about any missing or unclear information. | Page 8 |
| Study risk of bias assessment | 11 | Specify the methods used to assess risk of bias in the included studies, including details of the tool(s) used, how many reviewers assessed each study and whether they worked independently, and if applicable, details of automation tools used in the process. | Page 8 |
| Effect measures | 12 | Specify for each outcome the effect measure(s) (e.g. risk ratio, mean difference) used in the synthesis or presentation of results. | Page 8, 9 |
| Synthesis methods | 13a | Describe the processes used to decide which studies were eligible for each synthesis (e.g. tabulating the study intervention characteristics and comparing against the planned groups for each synthesis (item #5)). | Page 8, 9 |
|  | 13b | Describe any methods required to prepare the data for presentation or synthesis, such as handling of missing summary statistics, or data conversions. | Page 9 |
|  | 13c | Describe any methods used to tabulate or visually display results of individual studies and syntheses. | Page 8 |
|  | 13d | Describe any methods used to synthesize results and provide a rationale for the choice(s). If meta-analysis was performed, describe the model(s), method(s) to identify the presence and extent of statistical heterogeneity, and software package(s) used. | eMethod 4 |
|  | 13e | Describe any methods used to explore possible causes of heterogeneity among study results (e.g. subgroup analysis, meta-regression). | Page 9 |
|  | 13f | Describe any sensitivity analyses conducted to assess robustness of the synthesized results. | Page 9 |
| Reporting bias assessment | 14 | Describe any methods used to assess risk of bias due to missing results in a synthesis (arising from reporting biases). | eResults 2 |
| Certainty assessment | 15 | Describe any methods used to assess certainty (or confidence) in the body of evidence for an outcome. | N/A |
| **RESULTS** | | |  |
| Study selection | 16a | Describe the results of the search and selection process, from the number of records identified in the search to the number of studies included in the review, ideally using a flow diagram. | Figure 1 |
|  | 16b | Cite studies that might appear to meet the inclusion criteria, but which were excluded, and explain why they were excluded. | eResult 1 |
| Study characteristics | 17 | Cite each included study and present its characteristics. | Table 1 |
| Risk of bias in studies | 18 | Present assessments of risk of bias for each included study. | eFigure 1 |
| Results of individual studies | 19 | For all outcomes, present, for each study: (a) summary statistics for each group (where appropriate) and (b) an effect estimate and its precision (e.g. confidence/credible interval), ideally using structured tables or plots. | Figure 2, 3 |
| Results of syntheses | 20a | For each synthesis, briefly summarize the characteristics and risk of bias among contributing studies. | eResults 2 |
|  | 20b | Present results of all statistical syntheses conducted. If meta-analysis was done, present for each the summary estimate and its precision (e.g. confidence/credible interval) and measures of statistical heterogeneity. If comparing groups, describe the direction of the effect. | Page 11, 12 |
|  | 20c | Present results of all investigations of possible causes of heterogeneity among study results. | eResults 3,  eRestuls 5 |
|  | 20d | Present results of all sensitivity analyses conducted to assess the robustness of the synthesized results. | Page 11, 12 |
| Reporting biases | 21 | Present assessments of risk of bias due to missing results (arising from reporting biases) for each synthesis assessed. | Figure 2  Figure 3 |
| Certainty of evidence | 22 | Present assessments of certainty (or confidence) in the body of evidence for each outcome assessed. | N/A |
| **DISCUSSION** | | |  |
| Discussion | 23a | Provide a general interpretation of the results in the context of other evidence. | Page 13 |
|  | 23b | Discuss any limitations of the evidence included in the review. | Page 17, 18 |
|  | 23c | Discuss any limitations of the review processes used. | N/A |
|  | 23d | Discuss implications of the results for practice, policy, and future research. | Page 18 |
| **OTHER INFORMATION** | | |  |
| Registration and protocol | 24a | Provide registration information for the review, including register name and registration number, or state that the review was not registered. | Page 7 |
|  | 24b | Indicate where the review protocol can be accessed, or state that a protocol was not prepared. | Page 7 |
|  | 24c | Describe and explain any amendments to information provided at registration or in the protocol. | N/A |
| Support | 25 | Describe sources of financial or non-financial support for the review, and the role of the funders or sponsors in the review. | Page 18 |
| Competing interests | 26 | Declare any competing interests of review authors. | Page 2 |
| Availability of data, code and other materials | 27 | Report which of the following are publicly available and where they can be found: template data collection forms; data extracted from included studies; data used for all analyses; analytic code; any other materials used in the review. | eMethod 4 |

**Supplementary Method 2.** MOOSE checklist

| **Reporting Criteria** | **Reported (Yes/No)** | **Reported on Page No.** | **Brief description** |
| --- | --- | --- | --- |
| **Reporting of Background** |  |  |  |
| Problem definition | Yes | 6 | The interaction of PPI with chemotherapy in cancer patients has not been clarified. |
| Hypothesis statement | Yes | 7 | We are motivated to conduct this systematic review and meta-analysis to delve into PPI's influence on the effectiveness of chemotherapy in CRC patients. |
| Description of Study Outcome(s) | Yes | 8 | Survival outcomes of cancer patients treated with chemotherapy. |
| Type of exposure or intervention used | Yes | 8 | Cancer patients with and without baseline PPI use |
| Type of study design used | Yes | 8 | Randomized controlled trials, Prospective and retrospective cohort studies |
| Study population | Yes | 8 | Cancer patients treated with chemotherapy. |
| **Reporting of Search Strategy** |  |  |  |
| Qualifications of searchers (eg, librarians and investigators) | Yes | 7 | S.S.W, E.A, and M.A |
| Search strategy, including time period included in the synthesis and keywords | Yes | eMethod 3 | As shown in eMethod 3 |
| Effort to include all available studies, including contact with authors | Yes | N/A | We searched bibliographies of retrieved references.  It is unnecessary to contact authors as the data were publicly available. |
| Databases and registries searched | Yes | eMethod 3 | PubMed, Embase, Cochrane library, and Medline |
| Search software used, name and version, including special features used (eg, explosion) | Yes | eMethod 3 | Endnote X 9.3 was used to manage reference |
| Use of hand searching (eg, reference lists of obtained articles) | Yes | eMethod 3 | We searched bibliographies of retrieved references. |
| List of citations located and those excluded, including justification | Yes | eResult 1 | As shown in eResult 1 |
| Method for addressing articles published in languages other than English | Yes | N/A | We used translation software for addressing articles published in languages. |
| Method of handling abstracts and unpublished studies |  |  | We included conference abstract. |
| Description of any contact with authors | Yes | N/A | It is unnecessary to contact authors as the data were publicly available. |
| **Reporting of Methods** |  |  |  |
| Description of relevance or appropriateness of studies assembled for assessing the hypothesis to be tested | Yes | 8 | (1) randomized controlled trials, prospective or retrospective cohort studies;  (2) studies involving adult patients aged over 18 with cancers receiving ICI or chemotherapy;  (3) studies reporting at least one comparative survival outcome |
| Rationale for the selection and coding of data (eg, sound clinical principles or convenience) | Yes | 8 | Two investigators (T.H.W and Y.S.L) independently extracted relevant information from eligible articles. |
| Documentation of how data were classified and coded (eg, multiple raters, blinding, and interrater reliability) | Yes | 8 | Details of extraction are in page 8. |
| Assessment of confounding (eg, comparability of cases and controls in studies where appropriate | Yes | 8 | Two investigators (Y.C and R.B) independently completed a critical appraisal of included literature by using the Cochrane Risk of Bias tool 2.0^15^ for RCTs, and the Risk Of Bias In Non-randomized Studies of Interventions (ROBINS-I)^16^ tool for non-RCTs. |
| **Reporting Criteria** |  |  |  |
| Assessment of study quality, including blinding of quality assessors; stratification or regression on possible predictors of study results | Yes | 8 | ROBINS-I was applied. |
| Assessment of heterogeneity | Yes | 9 | Heterogeneity was assessed using I^2^ statistics proposed by Higgins and Thompson. |
| Description of statistical methods (eg, complete description of fixed or random effects models, justification of whether the chosen models account for predictors of study results, dose-response models, or cumulative meta-analysis) in sufficient detail to be replicated | Yes | eMethod 4 | We performed meta-analysis using random-effects model and meta-regression was conducted using a random-effects model. RStudio’s ‘‘metafor’’ package was used for all analyses. |
| Provision of appropriate tables and graphics | Yes | Figures and Tables | We included tables for illustrating details of included the studies and figures demonstrating a flow chart of study identification and the results of the meta-analyses. |
| **Reporting of Results** |  |  |  |
| Table giving descriptive information for each study included | Yes | Table 1 | Details are in Table 1 |
| Results of sensitivity testing (eg, subgroup analysis) | Yes | 11, 12 | We performed subgroup analysis based on different treatment modalities and cancer types. |
| Indication of statistical uncertainty of findings | Yes | 11, 12 | 95% confidence intervals and I^2^ values were presented with all effect estimates |
| **Reporting of Discussion** |  |  |  |
| Quantitative assessment of bias (eg, publication bias) | Yes | Figure 2, 3 | As shown in Figure 2, 3 |
| Justification for exclusion (eg, exclusion of non–English-language citations) | Yes | N/A | Studies were excluded based on the pre-specified eligibility criteria in Method. |
| Assessment of quality of included studies | Yes | eResult 2 | As shown in eResult 2 |
| Reporting of Conclusions |  |  |  |
| Consideration of alternative explanations for observed results | Yes | 13-18 | Elaborated in Discussion |
| Generalization of the conclusions (ie, appropriate for the data presented and within the domain of the literature review) | Yes | 18 | The use of PPI has little survival influence on CRC patients treated with capecitabine combination therapy. Conversely, both FOLFOX-treated and FOLFIRI-treated patients taking concomitant PPI trended toward both higher all-cause mortality and disease progression, however, with considerable heterogeneity. |
| Guidelines for future research | Yes | 18 | Future high-quality prospective studies investigating PPI use and FU-based regimens are warranted. |
| Disclosure of funding source | Yes | N/A | N/A |

**Supplementary Method 3.** Search strategy

***PubMed (30229)***

((PPI) or (proton pump inhibitor) or (gastric acid suppressants) or (esomeprazole) or (omeprazole) or (pantoprazole) or (rabeprazole) or (lansoprazole) or (dexlansoprazole) or (comedication) or (concomitant medication) or (prognostic factors) or (predictor)) and ((chemotherapeutic agent) or (chemotherapy) or (fluoropyrimidines) or (fluorouracil) or (fu) or (irinotecan) or (irinotecan) or (oxaliplatin) or (bevacizumab) or (ramucirumab) or (cetuximab) or (panitumumab) or (aflibercept) or (VEGF) or (EGFR) or (epidermal growth factor receptor) or (vascular endothelial growth factor) or (regorafenib) or (trifluridine-tipiracil) or (TAS-102) or (capecitabine) or (S-1) or (tegafur-uracil) or (UFT) or (FOLFIRI) or (FOLFOX) or (XELOX) or (Douillard)) and ((colorectal cancer) or (colorectal malignant neoplasm) or (colorectal carcinoma) or (colorectal malignancy) or (colon cancer) or (colon malignant neoplasm) or (colon carcinoma) or (colon malignancy) or (rectal cancer) or (rectal malignant neoplasm) or (rectal carcinoma) or (rectal malignancy))

***Embase***

| Search Number | Search Description | Numbers of results |
| --- | --- | --- |
| 1 | ((PPI) or (proton pump inhibitor) or (gastric acid suppressants) or (esomeprazole) or (omeprazole) or (pantoprazole) or (rabeprazole) or (lansoprazole) or (dexlansoprazole) or (comedication) or (concomitant medication) or (prognostic factors) or (predictor)):ti,ab,kw,de | 661758 |
| 2 | 'proton pump inhibitor'/exp | 90447 |
| 3 | ((chemotherapeutic agent) or (chemotherapy) or (fluoropyrimidines) or (fluorouracil) or (fu) or (irinotecan) or (irinotecan) or (oxaliplatin) or (bevacizumab) or (ramucirumab) or (cetuximab) or (panitumumab) or (aflibercept) or (VEGF) or (EGFR) or (epidermal growth factor receptor) or (vascular endothelial growth factor) or (regorafenib) or (trifluridine-tipiracil) or (TAS-102) or (capecitabine) or (S-1) or (tegafur-uracil) or (UFT) or (FOLFIRI) or (FOLFOX) or (XELOX) or (Douillard)):ti,ab,kw,de | 1501588 |
| 4 | 'molecularly targeted therapy'/exp | 50504 |
| 5 | 'chemotherapy'/exp | 768274 |
| 6 | ((colorectal cancer) or (colorectal malignant neoplasm) or (colorectal carcinoma) or (colorectal malignancy) or (colon cancer) or (colon malignant neoplasm) or (colon carcinoma) or (colon malignancy) or (rectal cancer) or (rectal malignant neoplasm) or (rectal carcinoma) or (rectal malignancy)):ti,ab,kw,de | 492690 |
| 7 | 'colorectal cancer '/exp | 351653 |
| 8 | (#1 or #2 ) and (#3 or #4 or #5 ) and (#6 or #7) | 8521 |

***Medline***

| Search Number | Search Description | Numbers of results |
| --- | --- | --- |
| 1 | (PPI) or (proton pump inhibitor) or (gastric acid suppressants) or (esomeprazole) or (omeprazole) or (pantoprazole) or (rabeprazole) or (lansoprazole) or (dexlansoprazole) or (comedication) or (concomitant medication) or (prognostic factors) or (predictor) | 338814 |
| 2 | exp Proton Pump Inhibitors/ | 20697 |
| 3 | (chemotherapeutic agent) or (chemotherapy) or (fluoropyrimidines) or (fluorouracil) or (fu) or (irinotecan) or (irinotecan) or (oxaliplatin) or (bevacizumab) or (ramucirumab) or (cetuximab) or (panitumumab) or (aflibercept) or (VEGF) or (EGFR) or (epidermal growth factor receptor) or (vascular endothelial growth factor) or (regorafenib) or (trifluridine-tipiracil) or (TAS-102) or (capecitabine) or (S-1) or (tegafur-uracil) or (UFT) or (FOLFIRI) or (FOLFOX) or (XELOX) or (Douillard) | 791141 |
| 4 | Exp Drug Therapy/ | 1471690 |
| 5 | (Colorectal cancer) or (colorectal malignant neoplasm) or (colorectal carcinoma) or (colorectal malignancy) or (colon cancer) or (colon malignant neoplasm) or (colon carcinoma) or (colon malignancy) or (rectal cancer) or (rectal malignant neoplasm) or (rectal carcinoma) or (rectal malignancy) | 201212 |
| 6 | Exp Colorectal Neoplasms/ | 227149 |
| 7 | (#1 or #2 ) and (#3 or #4 ) and (#5 or #6) | 3028 |

Cochrane CENTRAL (535)

**
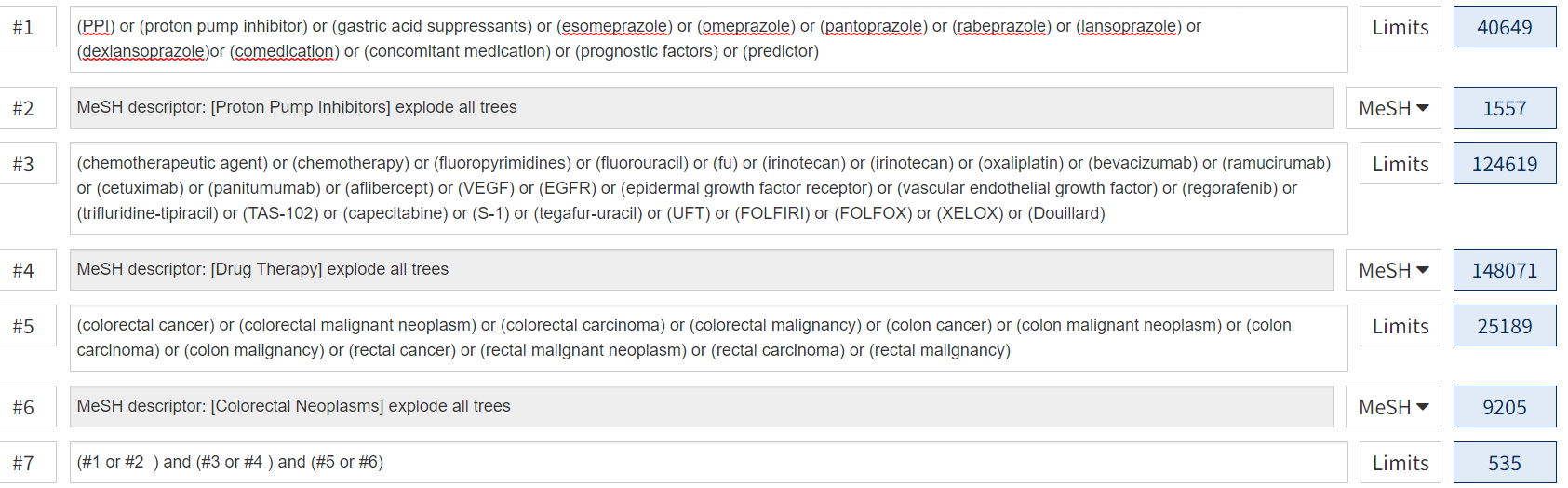
**

**Supplementary Method 4.** **Data synthesis**

We used Rstudio with meta, metafor, and netmeta packages to conduct statistical analysis:

【meta】package
Random-effects model:
Meta-analysis of binary outcome data (metabin)

Meta-analysis of continuous outcome data (metacont)

Generic inverse variance meta-analysis (metagen)

Several plots for meta-analysis: Forest plot (forest)
Funnel plot (funnel)

Exploring biases:
Egger’s test (metabias)

#Meta-analysis

un1<-read_xlsx('/Users/kychi/Desktop/ICI and PPI/Pairwise/Final analysis_unadjusted.xlsx', sheet = 'ICI_OS')

ICI_OS<-metagen(TE, seTE, studlab = study, data = un1, comb.fixed = F,

sm = 'HR', method.tau = 'REML', byvar = cancer)

forest(ICI_OS, layout = 'RevMan5', lab.e="With PPI", lab.c="Without PPI",

xlab="Favors PPI users Favors non-PPI users", ff.xlab="bold",

col.by="black", comb.fixed=F, col.diamond.random='red',

col.diamond.lines.random='red', col.square = 'blue', col.square.lines = 'blue',

print.byvar = F, test.subgroup.random = T, fontsize = 8, spacing = 0.75)

ICI_OS2<-metagen(TE, seTE, studlab = study, data = un1, comb.fixed = F,

sm = 'HR', method.tau = 'REML')

funnel (ICI_OS2, comb.random = F, contour.levels = c(0.9,0.95,0.99), col.contour = c("dark blue","blue","light blue"), ref = exp(ICI_OS$TE.fixed))

legend("topleft", c("p < 0.01", "0.01 < p < 0.05", "0.05 < p < 0.10", "p > 0.10"), fill=c("light blue","blue","dark blue", 'white'), bg = 'white')

ICIOS.bias <- metabias(ICI_OS2,method.bias="linreg",plotit=T)

ICIOS.bias

abline(h=c(0,-2,2), col = c("red","gray","gray"))

**Supplementary Result 1.** Studies that were excluded following full-text inspection

*No outcome of interest reported (n=21)*

1. Cesca M, Ruiz-Garcia E, Weschenfelder R, et al. P-197 The influence of proton pump inhibitor utilization on the pathological response of rectal cancer: A multicenter study by the Sociedad Latino Americana de Oncologia Gastrointestinal (SLAGO). Conference Abstract. *Annals of Oncology*. 2021;32:S167. doi:10.1016/j.annonc.2021.05.252

2. Matsuoka H, Ando K, Swayze EJ, et al. CTDSP1 inhibitor rabeprazole regulates DNA-PKcs dependent topoisomerase I degradation and irinotecan drug resistance in colorectal cancer. Article. *PLoS ONE*. 2020;15(8 July)doi:10.1371/journal.pone.0228002

3. Cheng V, Lemos MD, Hunter N, Badry N, Lemos JD. Concomitant use of capecitabine and proton pump inhibitors – Is it safe? Article. *Journal of Oncology Pharmacy Practice*. 2019;25(7):1705-1711. doi:10.1177/1078155219846952

4. de Man FM, Hussaarts K, de With M, et al. Influence of the Proton Pump Inhibitor Esomeprazole on the Bioavailability of Regorafenib: A Randomized Crossover Pharmacokinetic Study. *Clin Pharmacol Ther*. Jun 2019;105(6):1456-1461. doi:10.1002/cpt.1331

5. Eissele R, Arnold R. [Risk for developing tumors in therapy with the proton pump inhibitor omeprazole]. *Versicherungsmedizin*. Aug 1 1993;45(4):126-9. Risiko der Entwicklung von Tumoren unter der Therapie mit dem Protonenpumpen-Inhibitor Omeprazol.

6. Guisado Gil AB, Carrión Madroñal IM, Sánchez Gómez E, Santos Rubio MD. Detection and communication of concomitant use of capecitabine and proton pump inhibitors. Conference Abstract. *European Journal of Hospital Pharmacy*. 2020;27(SUPPL 1):A83. doi:10.1136/ejhpharm-2020-eahpconf.178

7. Han YM, Park JM, Kangwan N, et al. Role of proton pump inhibitors in preventing hypergastrinemia-associated carcinogenesis and in antagonizing the trophic effect of gastrin. Review. *Journal of Physiology and Pharmacology*. 2015;66(2):159-167.

8. Joo MK, Park JJ, Chun HJ. Additional Benefits of Routine Drugs on Gastrointestinal Cancer: Statins, Metformin, and Proton Pump Inhibitors. Review. *Digestive Diseases*. 2017;36(1):1-14. doi:10.1159/000480149

9. Lu ZN, Tian B, Guo XL. Repositioning of proton pump inhibitors in cancer therapy. Review. *Cancer Chemotherapy and Pharmacology*. 2017;80(5):925-937. doi:10.1007/s00280-017-3426-2

10. Miyamoto Y, Akiyama T, Kato R, et al. Prognostic Significance of Systemic Inflammation Indices by KRAS Status in Patients with Metastatic Colorectal Cancer. Article in Press. *Diseases of the colon and rectum*. 2022;doi:10.1097/DCR.0000000000002392

11. Morii Y, Fujimoto S, Nakahara R, et al. Effect of proton pump inhibitors on the development of hypomagnesemia induced by panitumumab. Article. *Die Pharmazie*. 2022;77(2):81-84. doi:10.1691/ph.2022.1988

12. Raoul JL, Edeline J, Gilabert M, Senellart H, Frenel JS. Proton pump inhibitors and cancers: A hazardous association? Review. *Bulletin du Cancer*. 2020;107(4):458-464. doi:10.1016/j.bulcan.2019.12.009

13. Sánchez RA, Nieto Gómez P, Valle Corpas M, Portillo Haro SFDA. Assesment of the interaction between proton pump inhibitors and capecitabine. Conference Abstract. *International Journal of Clinical Pharmacy*. 2020;42(1):256. doi:10.1007/s11096-019-00945-w

14. Sasaki T, Mori S, Kishi S, et al. Effect of Proton Pump Inhibitors on Colorectal Cancer. *Int J Mol Sci*. May 29 2020;21(11)doi:10.3390/ijms21113877

15. Schreiber V, Kitzmueller M, Poxhofer M, et al. Impact of co-administered drugs on drug monitoring of capecitabine in patients with advanced colorectal cancer. Article. *Anticancer Research*. 2014;34(7):3371-3376.

16. Sekido M, Fujita KI, Kubota Y, et al. Rabeprazole intake does not affect systemic exposure to capecitabine and its metabolites, 5'-deoxy-5-fluorocytidine, 5'-deoxy-5-fluorouridine, and 5-fluorouracil. *Cancer Chemother Pharmacol*. Jun 2019;83(6):1127-1135. doi:10.1007/s00280-019-03837-y

17. Spugnini E, Fais S. Proton pump inhibition and cancer therapeutics: A specific tumor targeting or it is a phenomenon secondary to a systemic buffering? Review. *Seminars in Cancer Biology*. 2017;43:111-118. doi:10.1016/j.semcancer.2017.01.003

18. Spugnini EP, Fais S. Drug repurposing for anticancer therapies. A lesson from proton pump inhibitors. Article. *Expert Opinion on Therapeutic Patents*. 2020;30(1):15-25. doi:10.1080/13543776.2020.1704733

19. Zeng X, Liu L, Zheng M, et al. Pantoprazole, an FDA-approved proton-pump inhibitor, suppresses colorectal cancer growth by targeting T-cell-originated protein kinase. *Oncotarget*. Apr 19 2016;7(16):22460-73. doi:10.18632/oncotarget.7984

20. Zheng M, Luan S, Gao S, et al. Proton pump inhibitor ilaprazole suppresses cancer growth by targeting T-cell-originated protein kinase. *Oncotarget*. Jun 13 2017;8(24):39143-39153. doi:10.18632/oncotarget.16609

21. Tvingsholm SA, Dehlendorff C, Østerlind K, Friis S, Jäättelä M. Proton pump inhibitor use and cancer mortality. Article. *International Journal of Cancer*. 2018;143(6):1315-1326. doi:10.1002/ijc.31529

*No PPI therapy (n= 16)*

1. Chen LJ, Schöttker B. Assessment of co-medication quality in older colorectal cancer patients at hospital discharge after tumor surgery and its associations with chemotherapy-related adverse drug reactions and 5-year survival. Conference Abstract. *Pharmacoepidemiology and Drug Safety*. 2021;30(SUPPL 1):75-76. doi:10.1002/pds.5305

2. Clémence B, Taieb J, Boulin M, et al. Impact of concomitant medications on disease free survival (DFS) and overall survival (OS) in patients from the PETACC8 study. Conference Abstract. *Annals of Oncology*. 2019;30:v207-v208. doi:10.1093/annonc/mdz246.026

3. Kobayashi Y, Shida D, Boku N, et al. Prognostic Factors of Bone Metastases from Colorectal Cancer in the Era of Targeted Therapy. Article in Press. *Diseases of the colon and rectum*. 2022;doi:10.1097/DCR.0000000000002270

4. Kondo A, Okano K, Kumamoto K, et al. Surgical management and outcomes of obstructive colorectal cancer in elderly patients: A multi-institutional retrospective study. Article in Press. *Surgery (United States)*. 2022;doi:10.1016/j.surg.2021.12.007

5. Labeda I, Lusikooy RE, Mappincara, et al. Colorectal cancer survival rates in Makassar, Eastern Indonesia: A retrospective Cohort Study. Article. *Annals of Medicine and Surgery*. 2022;74doi:10.1016/j.amsu.2021.103211

6. Lahoz S, Archilla I, Asensio E, et al. Copy-number intratumor heterogeneity increases the risk of relapse in chemotherapy-naive stage II colon cancer. Article in Press. *The Journal of pathology*. 2022;doi:10.1002/path.5870

7. Liang Y, Sun RL, Liu FY, Liu TT, Guan HQ, Tang DC. Anti-colorectal cancer mechanism of Astragali Radix-Curcumae Rhizoma- Paridis Rhizoma based on network pharmacology and experimental verification. Article. *Zhongguo Zhongyao Zazhi*. 2022;47(3):776-785. doi:10.19540/j.cnki.cjcmm.20211103.702

8. Matsumura K, Yamamura K, Miyamoto H, et al. Highly Advanced Colorectal Liver Metastases Successfully Treated with Fluorouracil Plus Leucovorin Monotherapy and Microwave Ablation. Article. *Anticancer Research*. 2022;42(3):1645-1651. doi:10.21873/anticanres.15641

9. Ohue M, Fujita S, Mizusawa J, et al. Preoperative and postoperative prognostic factors of patients with stage II/III lower rectal cancer without neoadjuvant therapy in the clinical trial (JCOG0212). Article. *Japanese Journal of Clinical Oncology*. 2022;52(2):114-121. doi:10.1093/jjco/hyab183

10. Picchetto A, Diana M, Swanström LL, et al. Upstaging nodal status in colorectal cancer using ex vivo fluorescence sentinel lymph node mapping: preliminary results. *Minim Invasive Ther Allied Technol*. Feb 2022;31(2):223-229. doi:10.1080/13645706.2020.1798464

11. Rong X, Liu H, Yu H, Zhao J, Wang J, Wang Y. Efficacy of apatinib combined with FOLFIRI in the first-line treatment of patients with metastatic colorectal cancer. Article in Press. *Investigational New Drugs*. 2022;doi:10.1007/s10637-021-01205-3

12. Shan L, Li T, Gu W, et al. Application of Prognostic Models Based on Psoas Muscle Index, Stage, Pathological Grade, and Preoperative Carcinoembryonic Antigen Level in Stage II-III Colorectal Cancer Patients Undergoing Adjuvant Chemotherapy. Article. *Journal of Oncology*. 2022;2022doi:10.1155/2022/6851900

13. Shang Y, Zhang Y, Liu J, et al. Decreased E2F2 Expression Correlates with Poor Prognosis and Immune Infiltrates in Patients with Colorectal Cancer. Article. *Journal of Cancer*. 2022;13(2):653-668. doi:10.7150/jca.61415

14. Shen F, Zhu Y, Wang F, et al. Clinical significance of circulating tumour cells and tumour marker detection in the chemotherapeutic evaluation of advanced colorectal cancer. Article. *Colorectal Disease*. 2022;24(1):68-76. doi:10.1111/codi.15939

15. Sun Y, Lin Y, Deng Y, et al. Identification of proteins associated with treatment response of neoadjuvant chemoradiotherapy in rectal mucinous adenocarcinoma by co-expression network analysis based on proteomic analysis. Article. *Journal of Proteomics*. 2022;254doi:10.1016/j.jprot.2021.104472

16. Torre A, Pereira H, Varanda J, et al. Survival and prognostic factors of resected colorectal liver metastasis after neoadjuvant chemotherapy. Conference Abstract. *European Journal of Surgical Oncology*. 2022;48(2):e114. doi:10.1016/j.ejso.2021.12.191

Supplementary Result 2. The Risk Of Bias In Non-randomized Studies – of Interventions (ROBINS-I) assessment tool

We used the ROBINS-I template provided by ROBINS-I detailed guidance (2016) to steer us toward the critical appraisal of included non-RCTs in our systematic review with meta-analysis regarding our review question. The details are presented in the following sections.

# ROBINS-I tool (Stage I): At protocol stage

## Specify the review question

| Participants | *Cancer patients receiving immune checkpoint inhibitors* |
| --- | --- |
| Experimental intervention | *With baseline proton pump inhibitors* |
| Comparator | *Without baseline proton pump inhibitors* |
| Outcomes | *Overall survival and progression-free survival between users and non-users* |

## List the confounding domains relevant to all or most studies

| *Age, ECOG-PS, PDL-1 expression, peptic ulcer disease, gastroesophageal reflux, coronary artery disease, first line treatment* |
| --- |

## List co-interventions that could be different between intervention groups and that could impact on outcomes

| *nil* |
| --- |

# ROBINS-I tool (Stage II): For each study

## Specify a target randomized trial specific to the study

| Design | Individually randomized / Cluster randomized / Matched (e.g. cross-over) |
| --- | --- |
| Participants | *Colorectal cancer patients receiving chemotherapy* |
| Experimental intervention | *With concomitant proton pump inhibitors or Histamine-2 blockers* |
| Comparator | *Without concomitant proton pump inhibitors or Histamine-2 blockers* |

## Is your aim for this study…?

| 🖙 | to assess the effect of *assignment to* intervention |
| --- | --- |
| 🖙 | to assess the effect of *starting and adhering to* intervention |

## Specify the outcome

Specify which outcome is being assessed for risk of bias (typically from among those earmarked for the Summary of Findings table). Specify whether this is a proposed benefit or harm of intervention.

| *Overall survival and progression-free survival* |
| --- |

## Specify the numerical result being assessed

In case of multiple alternative analyses being presented, specify the numeric result (e.g. RR = 1.52 (95% CI 0.83 to 2.77) and/or a reference (e.g. to a table, figure or paragraph) that uniquely defines the result being assessed.

| *Hazard Ratios* |
| --- |

## Preliminary consideration of confounders

Complete a row for each important confounding domain (i) listed in the review protocol; and (ii) relevant to the setting of this particular study, or which the study authors identified as potentially important.

#### “Important” confounding domains are those for which, in the context of this study, adjustment is expected to lead to a clinically important change in the estimated effect of the intervention. “Validity” refers to whether the confounding variable or variables fully measure the domain, while “reliability” refers to the precision of the measurement (more measurement error means less reliability).

| **(i) Confounding domains listed in the review protocol** | | | | |
| --- | --- | --- | --- | --- |
| Confounding domain | Measured variable(s) | Is there evidence that controlling for this variable was unnecessary?* | Is the confounding domain measured validly and reliably by this variable (or these variables)? | OPTIONAL: Is failure to adjust for this variable (alone) expected to favour the experimental intervention or the comparator? |
| Baseline confounding | Age | Yes | Yes | Favour comparator |
|  | ECOG-PS | Yes | Yes | Favour comparator |
|  | PDL-1 expression | Yes | No information | No information |
|  | Peptic ulcer disease | Yes | Yes | Favour comparator |
|  | Gastroesophageal reflux | Yes | Yes | Favour comparator |
|  | Coronary artery disease | Yes | Yes | Favour comparator |

| **(ii) Additional confounding domains relevant to the setting of this particular study, or which the study authors identified as important** | | | | |
| --- | --- | --- | --- | --- |
| Confounding domain | Measured variable(s) | Is there evidence that controlling for this variable was unnecessary?* | Is the confounding domain measured validly and reliably by this variable (or these variables)? | OPTIONAL: Is failure to adjust for this variable (alone) expected to favour the experimental intervention or the comparator? |
| Baseline confounding | First line treatment | No | Yes | No information |

* In the context of a particular study, variables can be demonstrated not to be confounders and so not included in the analysis: (a) if they are not predictive of the outcome; (b) if they are not predictive of intervention; or (c) because adjustment makes no or minimal difference to the estimated effect of the primary parameter. Note that “no statistically significant association” is not the same as “not predictive”.

## Preliminary consideration of co-interventions

Complete a row for each important co-intervention (i) listed in the review protocol; and (ii) relevant to the setting of this particular study, or which the study authors identified as important.

#### “Important” co-interventions are those for which, in the context of this study, adjustment is expected to lead to a clinically important change in the estimated effect of the intervention.

| **(i) Co-interventions listed in the review protocol** | | |
| --- | --- | --- |
| Co-intervention | Is there evidence that controlling for this co-intervention was unnecessary (e.g. because it was not administered)? | Is presence of this co-intervention likely to favour outcomes in the experimental intervention or the comparator |
| Nil | - | - |

| **(ii) Additional co-interventions relevant to the setting of this particular study, or which the study authors identified as important** | | |
| --- | --- | --- |
| Co-intervention | Is there evidence that controlling for this co-intervention was unnecessary (e.g. because it was not administered)? | Is presence of this co-intervention likely to favour outcomes in the experimental intervention or the comparator |
| Nil | - | - |

**•**Justification of risk of bias of each study in each domain

| Study | Description | Risk of bias |
| --- | --- | --- |
| Domain 1: Bias due to confounding | | |
| Wong 2019 | There was a lack of details regarding the baseline demographics between PPI users and PPI non-users. However, the study did conduct multivariate analysis regarding baseline use of PPI. | Moderate |
| Wang 2017 | The study was not only scarce of baseline characteristics between PPI users and PPI non-users but of adjusted analysis for significant variables. | Serious |
| Kitazume 2022 | There was a lack of details regarding the baseline demographics between PPI users and PPI non-users. However, the study did conduct multivariate analysis regarding baseline use of PPI. | Moderate |
| Bridoux 2022 | The baseline characteristics of PPI users and PPI non-users were not matched and only univariate outcome was reported | Serious |
| **Domain 2: Bias in selection of participants into the study** | | |
| **Domain 3: Bias in classification of interventions** | | |
| Sun 2016 | The study obtained the use of PPI by reviewing the clinical records; however, PPI use window was not defined. | Moderate |
| Wong 2019 | The study obtained the use of PPI by reviewing the clinical records; however, PPI use window was not defined. | Moderate |
| Bridoux 2022 | The study obtained the use of PPI by reviewing the clinical records; however, PPI use window was not defined. | Moderate |

| **Domain 4: Bias due to deviations from intended interventions** | | | |
| --- | --- | --- | --- |
|  | **If your aim for this study is to assess the effect of assignment to intervention, answer questions 4.1 and 4.2** | |  |
|  | 4.1. Were there deviations from the intended intervention beyond what would be expected in usual practice? |  | N |
|  | **Risk of bias judgement** |  | Low |
| **Domain 5: Bias due to missing data** | | | |
|  | 5.1 Were outcome data available for all, or nearly all, participants? |  | Y |
|  | 5.2 Were participants excluded due to missing data on intervention status? |  | N |
|  | 5.3 Were participants excluded due to missing data on other variables needed for the analysis? |  | N |
|  | **Risk of bias judgement** |  | Low |
| **Domain 6: Bias in measurement of outcomes** | | | |
|  | 6.1 Could the outcome measure have been influenced by knowledge of the intervention received? |  | N |
|  | 6.2 Were outcome assessors aware of the intervention received by study participants? |  | Y |
|  | 6.3 Were the methods of outcome assessment comparable across intervention groups? |  | Y |
|  | 6.4 Were any systematic errors in measurement of the outcome related to intervention received? |  | N |
|  | **Risk of bias judgement** |  | Low |
| **Domain 7: Bias in selection of the reported result** | | | |
|  | Is the reported effect estimate likely to be selected, on the basis of the results, from... |  |  |
|  | 7.1. ... multiple outcome *measurements* within the outcome domain? |  | N |
|  | 7.2 ... multiple *analyses* of the intervention-outcome relationship? |  | N |
|  | 7.3 ... different *subgroups*? |  | N |
|  | **Risk of bias judgement** |  | Low |

The risk of bias was evaluated as low through domain 4 to domain 7 across included

**Supplementary Figure 1.** Visual summary of ROBINS-I for included studies *
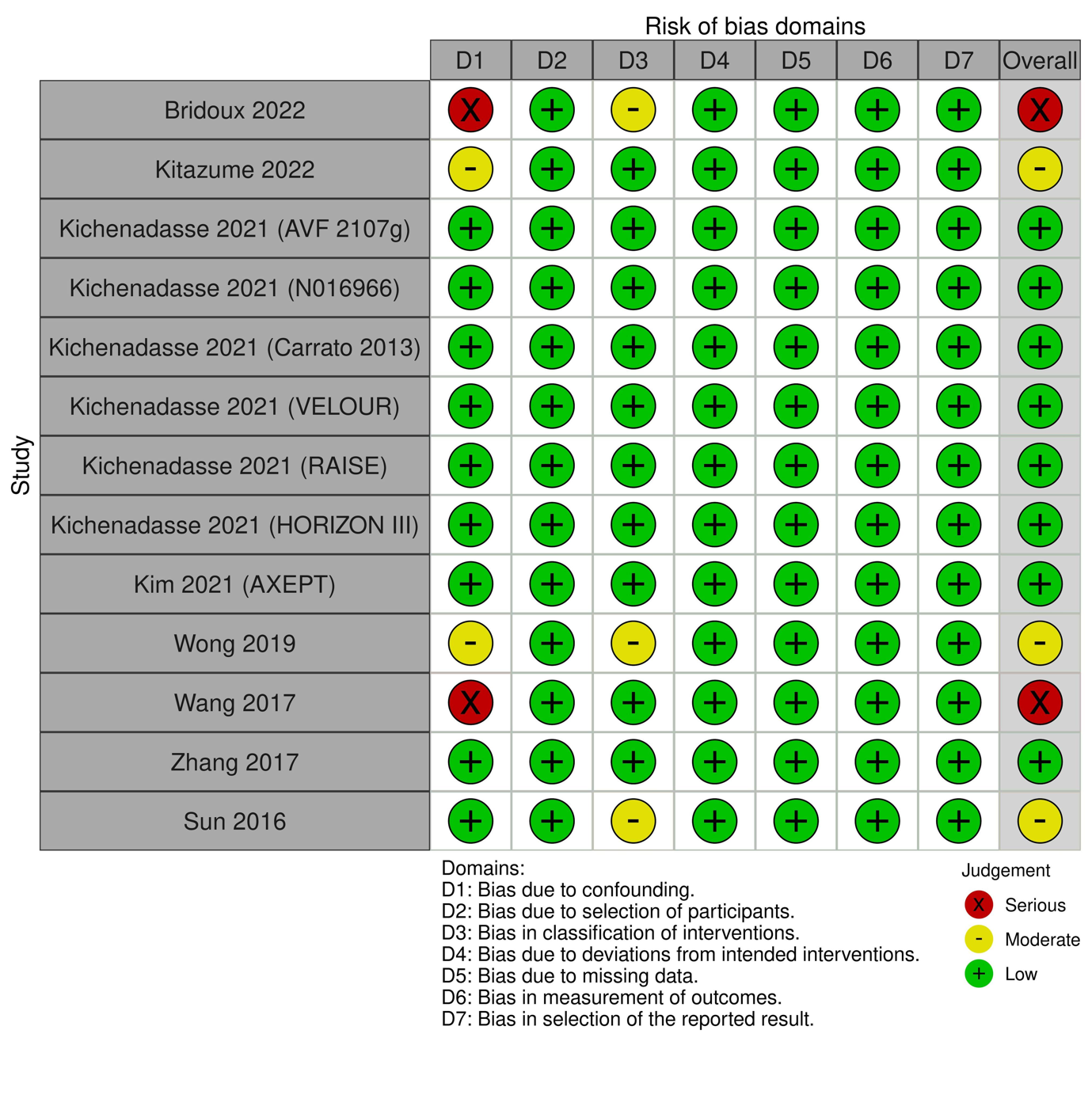
*

**Supplementary Results 3.** Assessment of publication bias in patients taking capecitabine-based chemotherapy


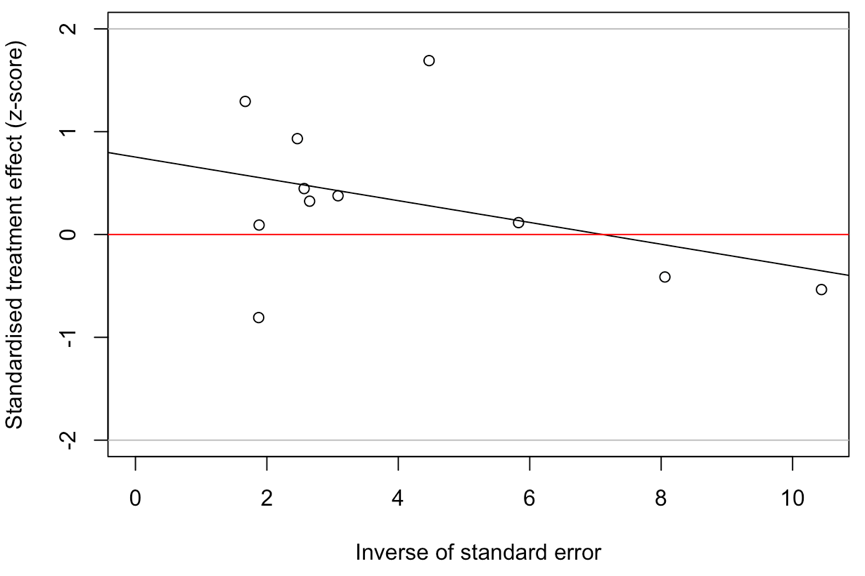


Linear regression test of funnel plot asymmetry
Test result: t = 1.88, df = 9, p-value = 0.0927

Sample estimates:

bias se.bias. intercept se.intercept

0.7529 0.4003 -0.1060 0.0813

Details:

- multiplicative residual heterogeneity variance (tau^2 = 0.5476)

- predictor: standard error

- weight: inverse variance

- reference: Egger et al. (1997), BMJ

**
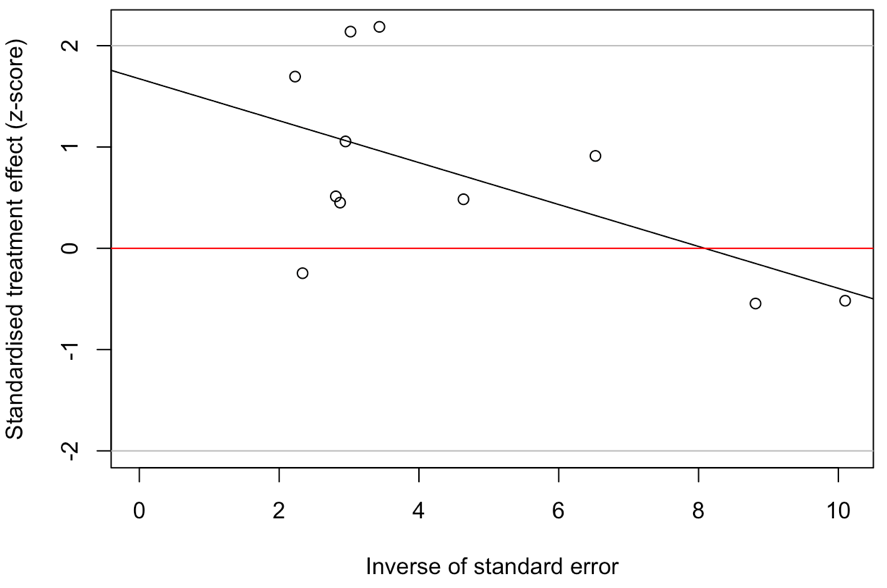
**

Linear regression test of funnel plot asymmetry

Test result: t = 3.33, df = 9, p-value = 0.0088

Sample estimates:

bias se.bias intercept se.intercept

1.6743 0.5031 -0.2070 0.0964

Details:

- multiplicative residual heterogeneity variance (tau^2 = 0.6977)

- predictor: standard error

- weight: inverse variance

- reference: Egger et al. (1997), BMJ


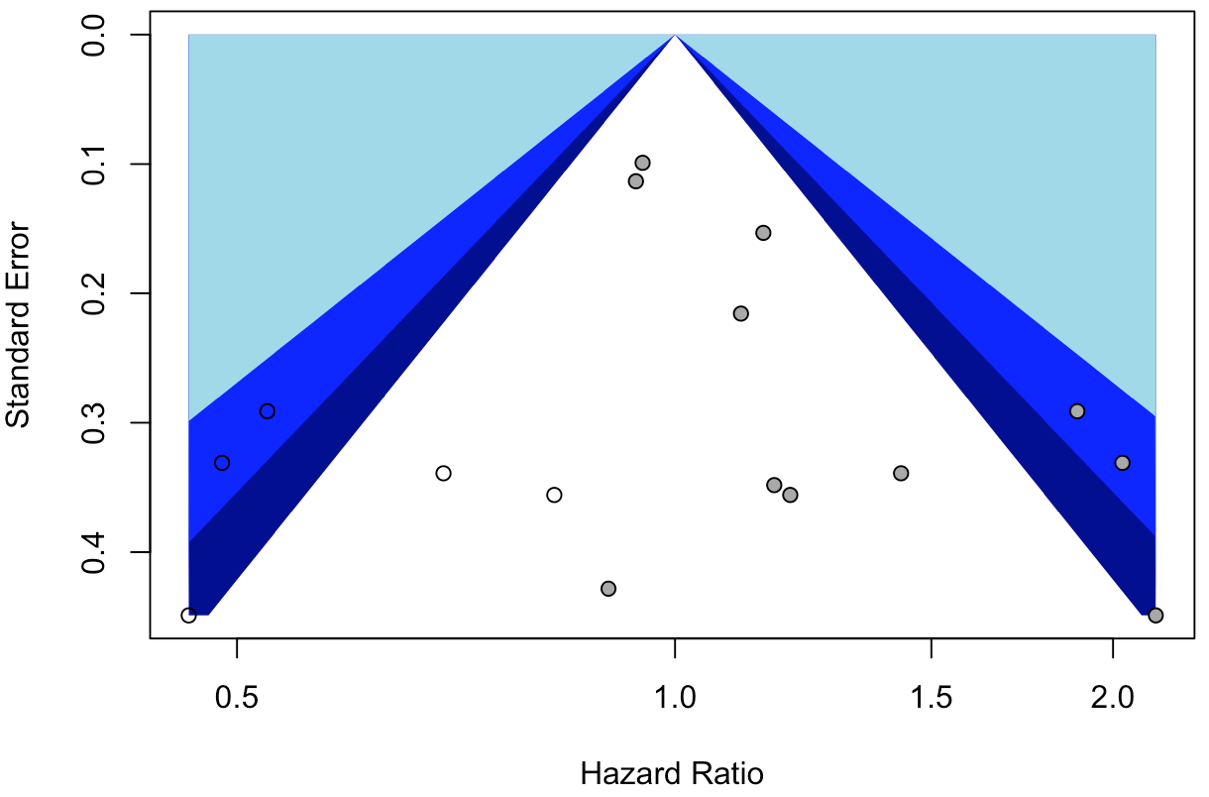


**Supplementary Figure 2.** Funnel plot of PFS after the trim-and-fill analysis with 5 fictive studies being imputed

Number of studies combined: k = 16 (with 5 added studies)

HR 95%-CI z p-value

Random effects model 1.0112 [0.8384; 1.2195] 0.12 0.9074

Quantifying heterogeneity:

tau^2 = 0.0653 [0.0066; 0.4137]; tau = 0.2556 [0.0815; 0.6432]

I^2 = 49.1% [9.4%; 71.4%]; H = 1.40 [1.05; 1.87]

Test of heterogeneity:

Q d.f. p-value

29.49 15 0.0139

Details on meta-analytical method:

- Inverse variance method

- Restricted maximum-likelihood estimator for tau^2

- Q-profile method for confidence interval of tau^2 and tau

- Trim-and-fill method to adjust for funnel plot asymmetry

**Supplementary Result 4.** Prespecified sensitivity analyses of patients taking capecitabine-based chemotherapy


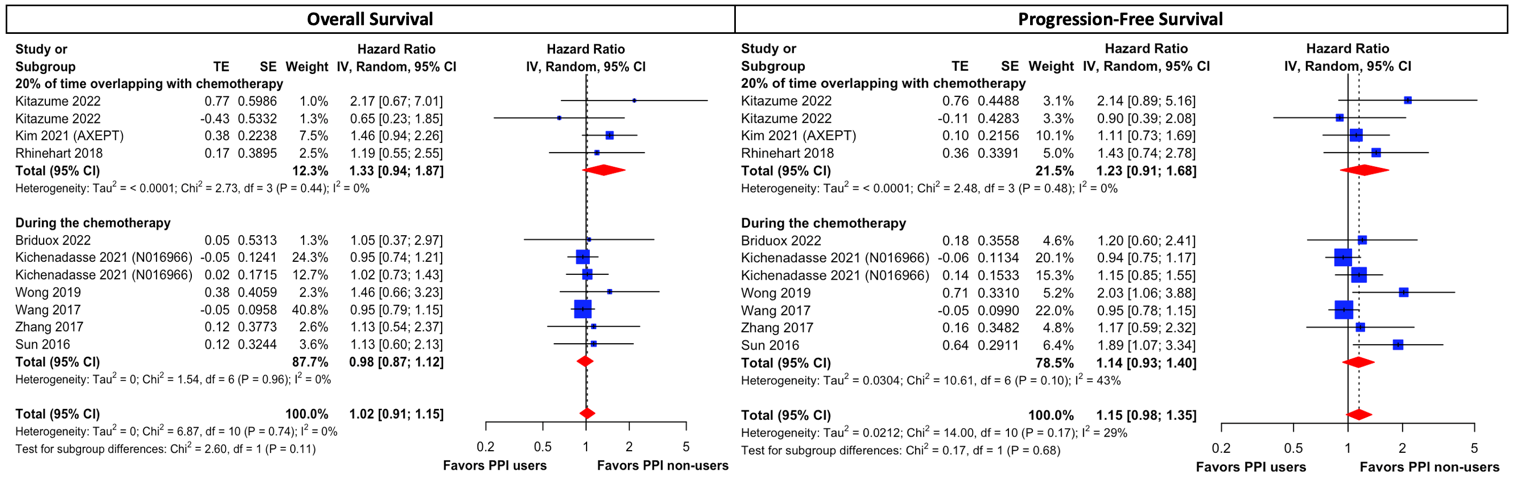


**Supplementary Figure 3.** Forest plot for the subgroup analysis based on PPI administration window. Both overall survival and progression-free survival were comparable between PPI users and non-users in patients receiving PPI during chemotherapy and in patients receiving PPI at least 20% during treatment period.


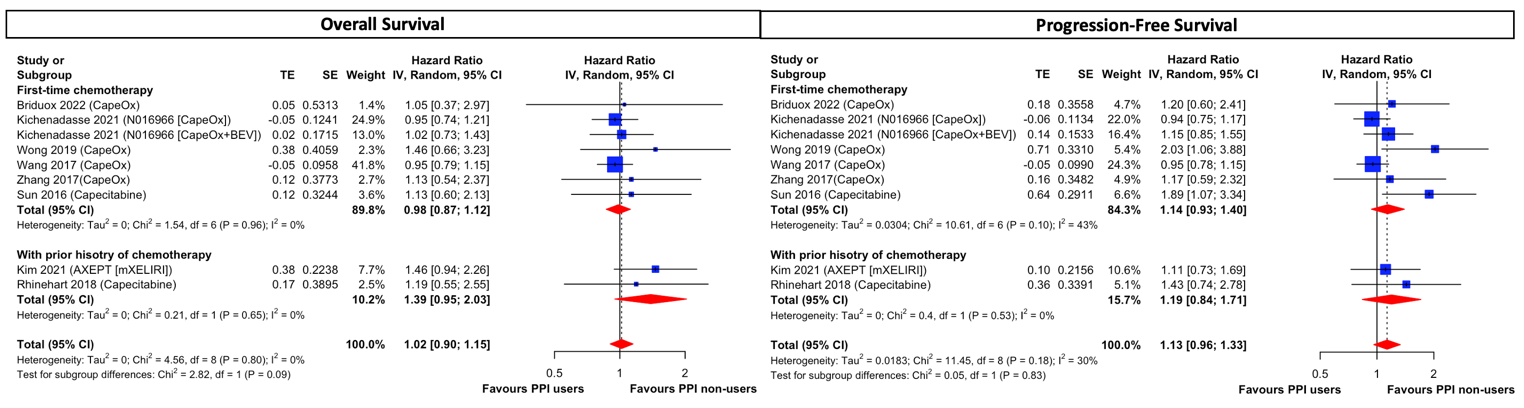


**Supplementary Figure 4.** Forest plot for the subgroup analysis based on history of prior capecitabine-based chemotherapy in colorectal cancer. No significant difference between PPI users and non-users in overall survival and progression-free survival for patients receiving first line treatment and patients receiving non-first line treatment.

**Supplementary Result 5.** Assessment of publication bias in patients taking fluorouracil-based chemotherapy


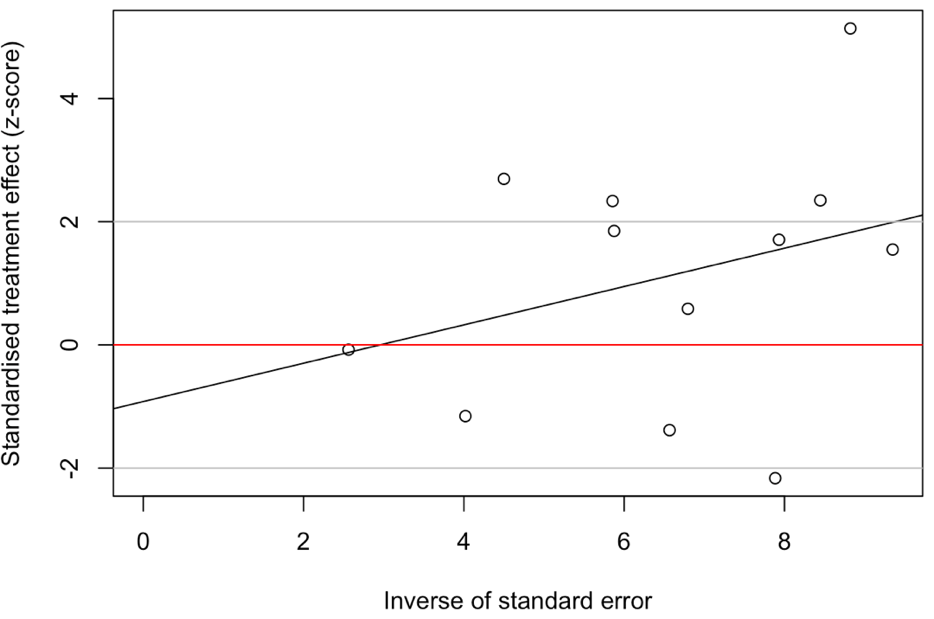

Linear regression test of funnel plot asymmetry

Test result: t = -0.45, df = 10, p-value = 0.6598

Sample estimates:

bias se.bias intercept se.intercept

-0.9201 2.0287 0.3112 0.2963

Details:

- multiplicative residual heterogeneity variance (tau^2 = 4.1970)

- predictor: standard error

- weight: inverse variance

- reference: Egger et al. (1997), BMJ


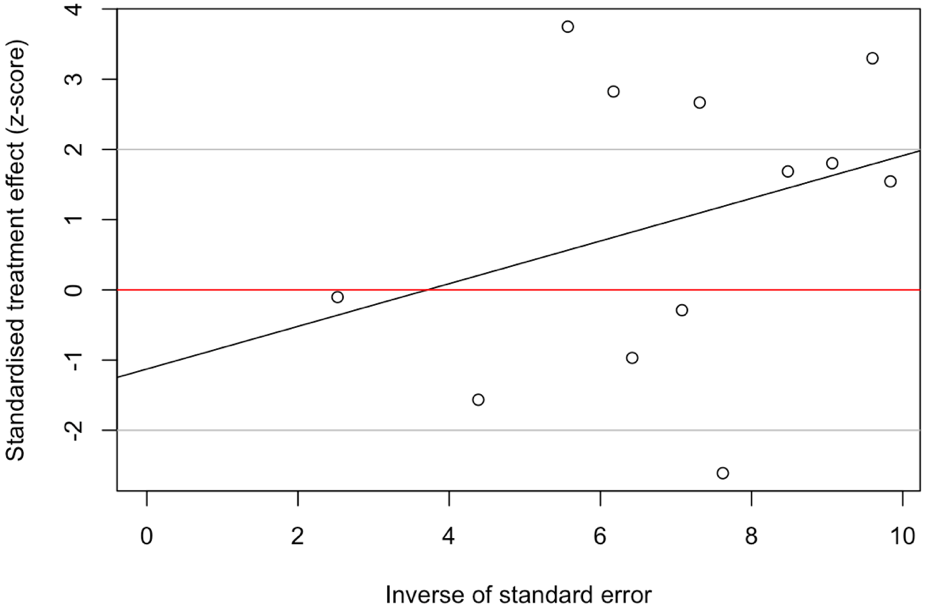


Linear regression test of funnel plot asymmetry

Test result: t = -0.54, df = 10, p-value = 0.5999

Sample estimates:

bias se.bias intercept se.intercept

-1.1271 2.0808 0.3040 0.2848

Details:

- multiplicative residual heterogeneity variance (tau^2 = 4.1926)

- predictor: standard error

- weight: inverse variance

- reference: Egger et al. (1997), BMJ

**Supplementary Result 6.** Prespecified sensitivity analyses of patients taking fluorouracil-based chemotherapy


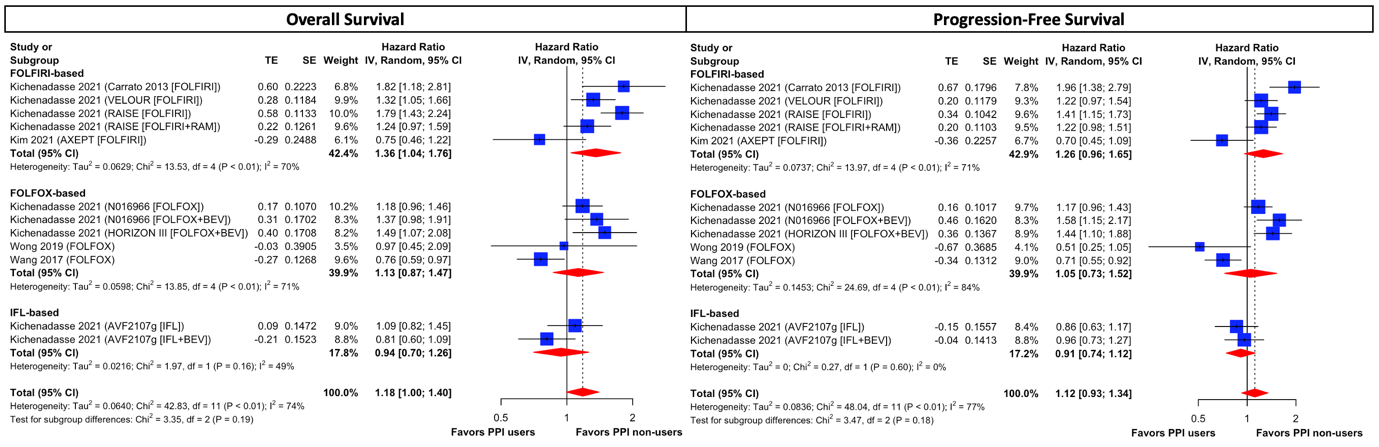


**eFigure 5.** Forest plot for the subgroup analysis based on regimen modifications of fluorouracil-based chemotherapy in colorectal cancer. FOLIRI-treated patients with concomitant PPI were associated with worsening survival outcomes, as compared to PPI non-users. On the other hand, both overall survival and progression-free survival were comparable between PPI users and non-users in patients receiving FOLFOX-based therapy and in patients receiving IFL-based therapy.

**Supplementary Result 7.** Post hoc exploratory analysis of patients taking fluorouracil-based chemotherapy


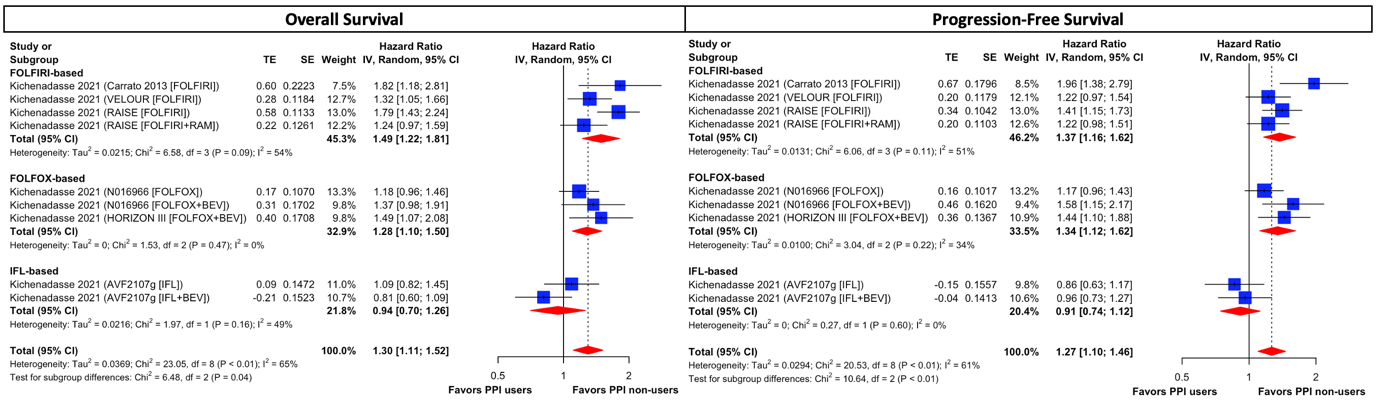


**eFigure 6.** Forest plot for the post hoc exploratory analysis based on regimen modifications of fluorouracil-based chemotherapy in colorectal cancer.

**Supplementary Result 8.** Comparative survival outcomes between PPI users and non-users using adjusted outcomes


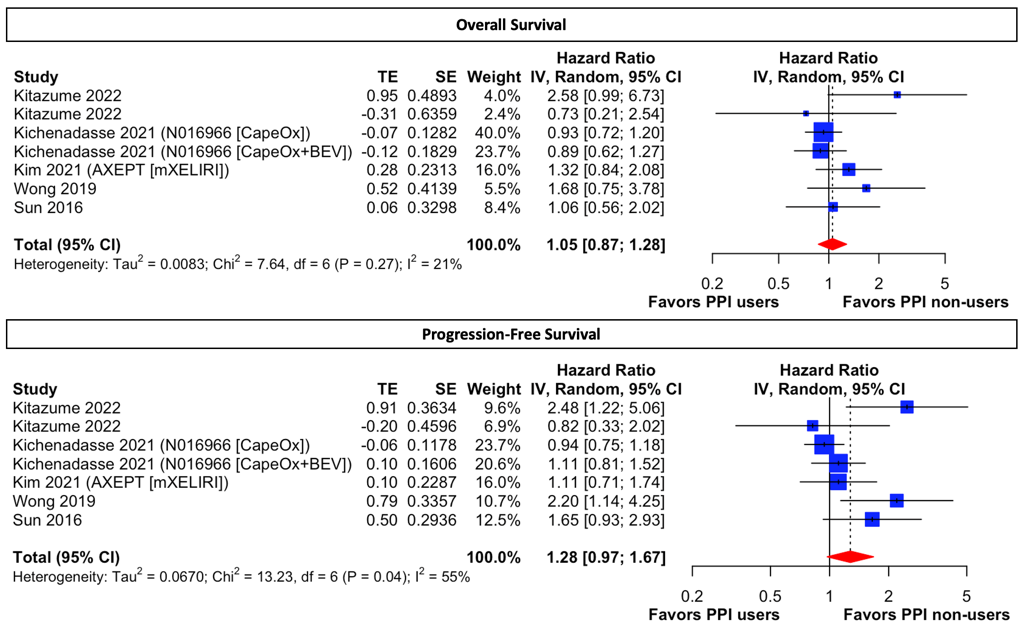


**Supplementary Figure 7**. Forest plot of patients taking capecitabine-based regimens using adjusted hazard ratios. Adjusted variables are available in eTable 3.


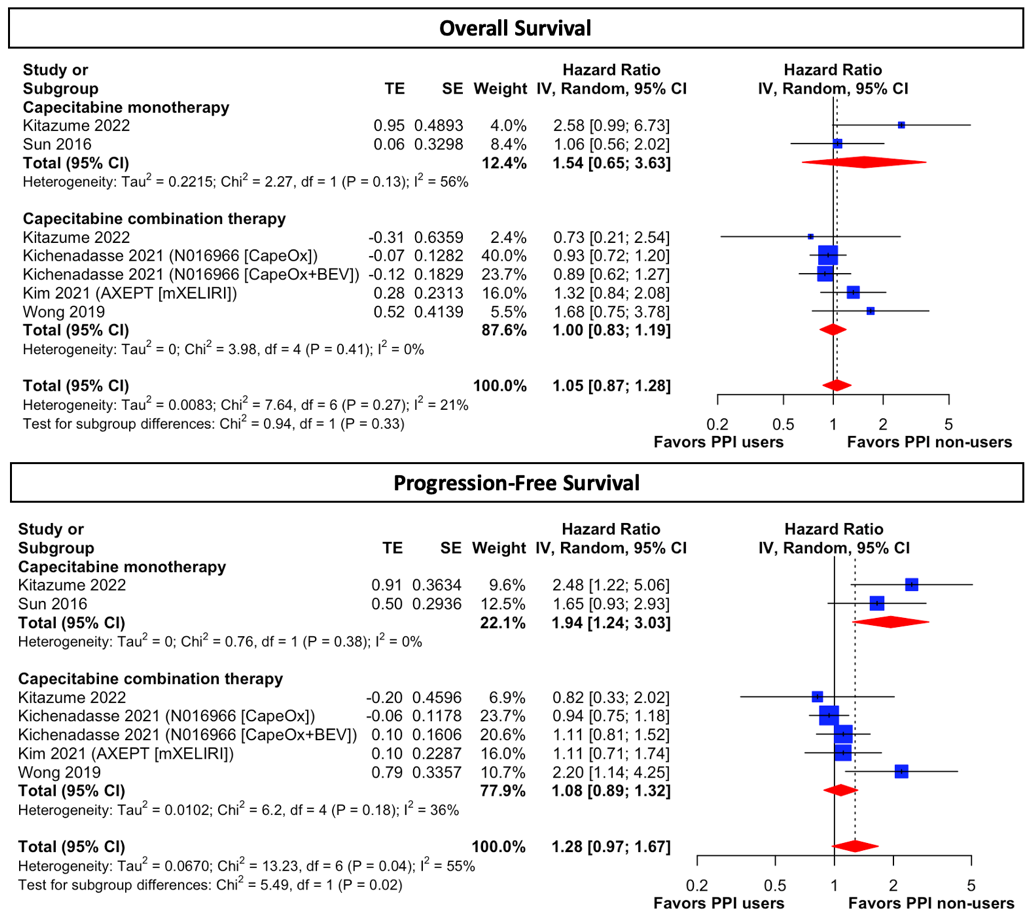


**Supplementary Figure 8.** Forest plot for subgroup analysis of regimen modifications in patients taking capecitabine-based regimens using adjusted hazard ratios. Adjusted variables are available in eTable 3.


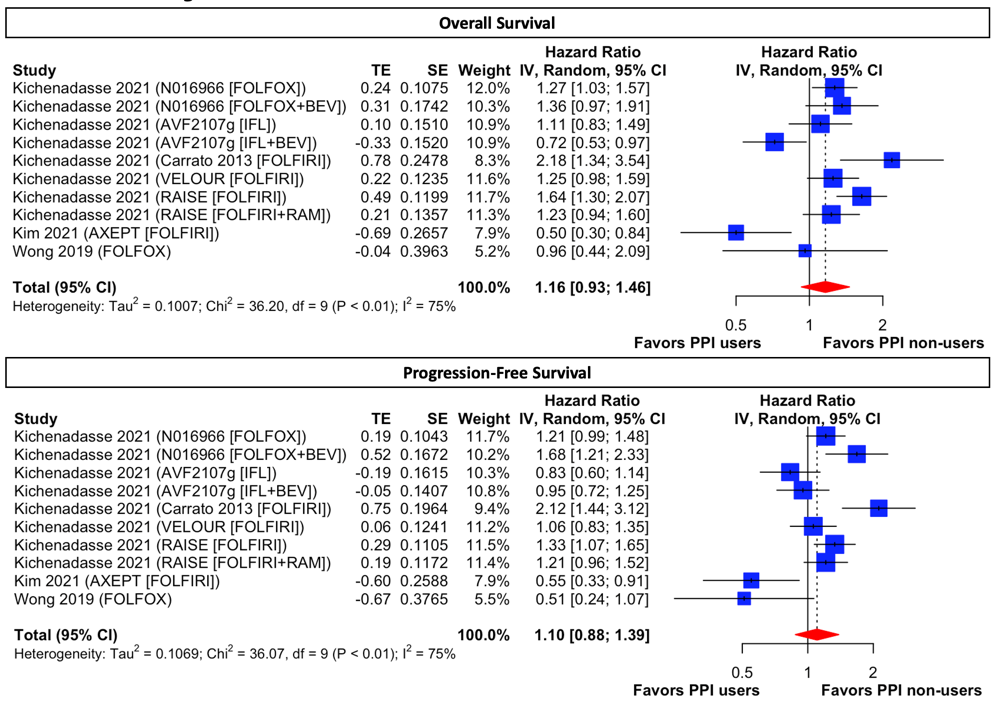


**Supplementary Figure 9.** Forest plot of patients taking fluorouracil-based regimens using adjusted hazard ratios. Adjusted variables are available in eTable 3.


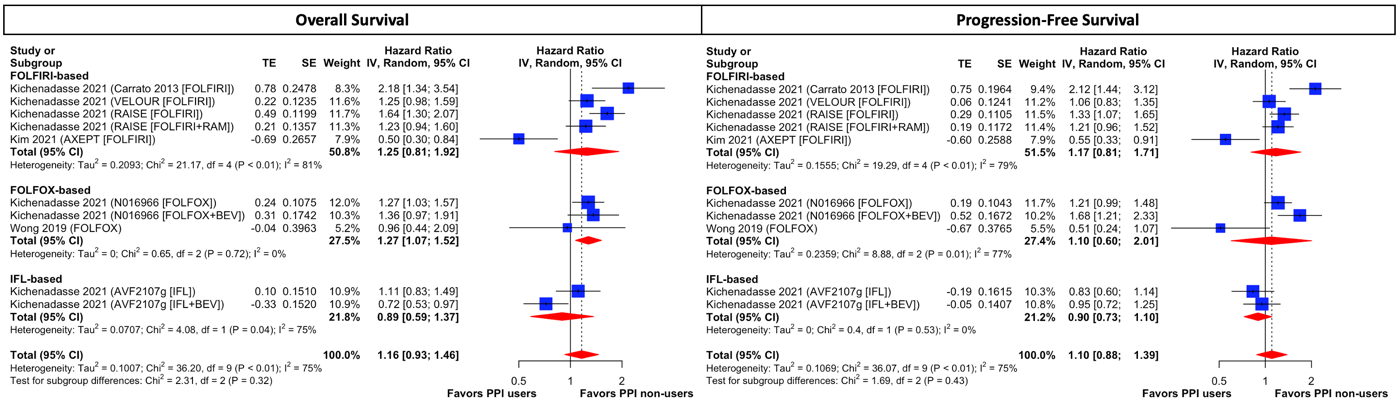


**Supplementary Figure 10**. Forest plot for subgroup analysis of regimen modifications in patients taking fluorouracil-based regimens using adjusted hazard ratios. Adjusted variables are available in eTable 3.


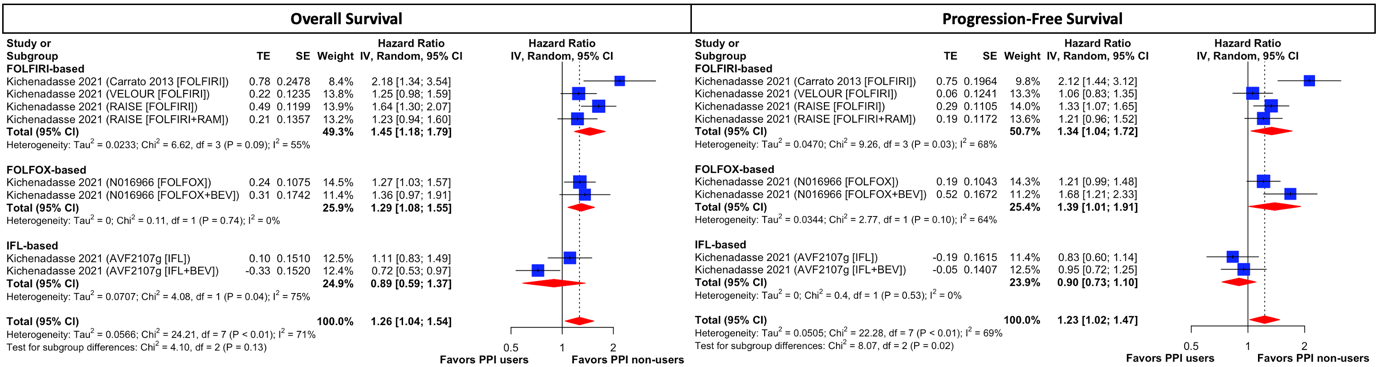


**Supplementary Figure 11.** Forest plot for post hoc exploratory analysis in patients taking fluorouracil-based regimens using adjusted hazard ratios. AXEPT was excluded in FOLFIRI-based regimen and Wong 2019 was removed in FOLFOX-based regimen. Adjusted variables are available in eTable 3.

| **Supplementary Table 1. Eligibility criteria of studies patients included in meta-analysis** | | |
| --- | --- | --- |
| Trial | Inclusion criteria | Exclusion criteria |
| AVF2107 | 1. Age>18, with histologically confirmed metastatic colorectal cancer (MCRC), bidimensional measurable lesions; 2. ECOG-PS 0-1, with a life expectancy of longer than 3 months; 3. Adequate hematologic, hepatic, and renal function (including urinary excretion of no more than 500 mg of protein per day). 4. Prior radiotherapy for MCRC was permitted if completed > 2 weeks before and random assignment 5. Major surgery was permitted if completed > 4 weeks before and random assignment | 1. Pregnant or breast-feeding women; 2. Known CNS metastases; 3. Cardiovascular disease; 4. Clinically detectable ascites; 5. Regular use of aspirin (more than 325 mg per day) or other nonsteroidal anti-inflammatory agents; 6. Preexisting bleeding diatheses or coagulopathy or the need for full-dose anticoagulation; 7. Prior chemotherapy or biologic therapy for metastatic disease |
| N016966 | 1. Age>18, with histologically confirmed MCRC, one or more uni-dimensionally measurable lesions; 2. ECOG-PS 0-1, with a life expectancy of longer than 3months; 3. No prior systemic therapy for MCRC; 4. Radiotherapy or surgery for MCRC was permitted if completed > 4 weeks before random assignment | 1. Pregnant or breast-feeding women; 2. Known CNS metastases; 3. Cardiovascular disease; 4. Clinically detectable ascites; 5. Use of full-dose anticoagulants or thrombolytics; 6. Serious nonhealing wound, ulcer, or bone fracture; 7. Clinically significant bleeding diathesis or coagulopathy; and 8. Proteinuria > 500 mg/24 hours. |
| Carrato 2013 | 1. Age ≥ 18, with histologically confirmed MCRC, with at least one measurable lesion as defined by RECIST criteria; 2. ECOG-PS 0-1; 3. Radiotherapy was permitted if full-field is completed > 4 weeks or limited field is completed > 2 weeks before random assignment 4. Major surgical procedure, open biopsy or significant traumatic injury was permitted if >4 weeks before randomization; | 1. Pregnant or lactating women; 2. Prior treatment with a VEGF, VEGFR or RTK inhibitor; 3. Cardiovascular diseases:  poorly controlled hypertension, coronary artery diseases, congestive heart failure, ongoing arrythmia, cerebrovascular accident, thromboembolism; 4. GI comorbidities:  peptic ulcer disease, infectious or inflammatory bowel disease, diverticulitis, unresolved bowel obstruction or chronic diarrhea, recent history of abdominal fistula, gastrointestinal perforation, or intra-abdominal abscess within 6 months prior to study enrollment 5. Known CNS metastases; 6. Use of full-dose anticoagulants; 7. History of clinically significant bleeding within the past 6 months, including gross hemoptysis or hematuria, or underlying coagulopathy; 8. Active infection, or on antiretroviral therapy for HIV disease. |
| VELOUR | 1. Age ≥ 18, with pathohistologically confirmed MCRC 2. ECOG PS 0-2; 3. Patients have documented progression while on or after completion of a single prior oxaliplatin-containing regimen; 4. Prior bevacizumab was permitted, but not prior irinotecan. 5. Major surgery was permitted if completed > 4 weeks before randomization | 1. Pregnant or lactating women; 2. Known prior malignancies or CNS metastases; 3. Severe acute or chronic medical condition that may have impaired the ability to participate in the study or interfered with the interpretation of results; 4. Poorly controlled hypertension or thromboembolism. |
| RAISE | 1. Age> 18, with pathologically confirmed MCRC, known KRAS exon 2 mutation (mutant or wild-type); 2. ECOG-PS 0-1; 3. Eligible patients had disease progression during or within 6 months of the last dose of first-line combination therapy with bevacizumab, oxaliplatin, and a fluoropyrimidine, and had received at least one cycle of the triplet therapy 4. Prior bevacizumab was permitted if completed > 4 weeks before random assignment 5. Prior chemotherapy was permitted if completed > 3 weeks before randomization | 1. Known CNS metastases; 2. Poorly controlled hypertension; 3. Any thromboembolism within 12 months before randomisation or during first-line therapy; 4. Grade 3–4 bleeding event, Grade 3 proteinuria, or bowel perforation during first-line therapy; 5. Grade 3–4 bleeding within 3 months before randomisation |
| Bridoux 2022 | 1. Age > 18 years, with diagnosis of locally advanced rectal adenocarcinoma; 2. Patients receiving CRT including capecitabine followed by surgery with or without adjuvant chemotherapy; 3. Patients who started receiving RT between January 1, 2004, and December 31, 2017. | 1. Patients with metastatic disease 2. Patients without rectal adenocarcinoma 3. Patients not receiving RT at the cancer center 4. Patients not receiving oral 5-FU 5. Patients not undergoing rectal surgery 6. Patients receiving first-line chemotherapy before CRT 7. Patients under tutorship or curatorship. |
| Kitazume 2022 | 1. Age ≥ 20 years with pathologically diagnosed stage II–III CRC and who had undergone curative surgery; 2. Patients who had received at least one course of adjuvant capecitabine monotherapy (2500 mg/m2 days 1–14 every 3 weeks) or CapeOX regimen (capecitabine 2000 mg/m2 days 1–14 and oxaliplatin 130 mg/m2 day 1 every 3 weeks) between January 2009 and December 2014 | 1. History of prior administration of capecitabine monotherapy or CapeOX regimen before the investigation period; 2. History of prior administration of any adjuvant chemotherapy, except for capecitabine monotherapy or CapeOX regimen; 3. History of prior administration of any neoadjuvant chemotherapy; 4. Concurrent radiotherapy during adjuvant chemotherapy; |
| Wong 2019 | 1. Age ≥ 18 years, were formally diagnosed with and received surgical treatment for colorectal cancer; 2. Patients who had stage II-III disease at the time of CapeOx or FOLFOX initiation; 3. Patients who received their first cycle of adjuvant CapeOx or FOLFOX from January 1, 2004 to December 31, 2013 | 1. Patients with in situ (stage 0) or localized (stage I) disease; 2. Patients with metastatic disease (stage IV) were excluded; 3. Patients receiving concurrent radiotherapy; 4. Patients who had missing information from their records |
| Rhinehart 2018 | 1. Age ≥ 18 years, had a diagnosis of colon or rectal cancer; 2. Patients were on capecitabine mono-therapy without concurrent radiation therapy during the study period; 3. Patients with a minimum follow-up of three weeks | N/A |
| Wang 2017 | 1. More than 18 years old 2. Patients did not receive surgical treatment | 1. Received concurrent radiotherapy |
| Zhang 2017 | 1. Patients who received the same neoadjuvant CRT followed by radical surgery | 1. Patients lacking detailed medical records; 2. Patients with a second tumor or distant metastasis |
| Sun 2016 | 1. Age ≥ 18 years, had a diagnosis of colon or rectal cancer and received their first dose of capecitabine between January 1st, 2008 and December 31, 2012. | 1. Patients had metastatic disease; 2. Patients received less than 3 cycles of therapy for reasons other than toxicity; 3. Patients received concurrent radiotherapy. |

| **Supplementary Table 2. Details of proton pump inhibitors** | | | | | | |
| --- | --- | --- | --- | --- | --- | --- |
| Included studies | PPI users,  n | Omeprazole, n (%) | Pantoprazole,  n (%) | Esomeprazole, n (%) | Lansoprazole, n (%) | Rabeprazole, n (%) |
| Bridoux 2022 | 25 | 6 (24) | 3 (12) | 14 (56) | 1 (4) | 1 (4) |
| Kitazume 2022 | 54 | 4 (7.4) | 0 (0) | 10 (18.5) | 30 (55.6) | 10 (18.5) |
| Wong 2019 | 99 | NA | NA | NA | NA | NA |
| Rhinehart 2018 | 18 | NA | NA | NA | NA | NA |
| Sun 2016 | 77 | NA | NA | NA | NA | NA |
| Wang 2017 | 474 | NA | NA | NA | NA | NA |
| Zhang 2017 | 63 | 63(100) | 0(0) | 0(0) | 0(0) | 0(0) |
| AXEPT | 49 | NA | NA | NA | NA | NA |
| HORIZON III | 87 | 36 (40.9) | 22 (24.9) | 10 (11.4) | 15 (17.0) | 5 (5.7) |
| N016966 | 327 | 115 (35.1) | 38 (11.6) | 34 (10.3) | 48 (14.6) | 8 (2.4) |
| AVF2107g | 159 | 46 (28.9) | 31 (19.4) | 29 (18.2) | 60 (37.7) | 10 (6.2) |
| Carrato 2013 | 43 | 22 (51.1) | 12 (2.9) | 6 (13.9) | 3 (6.9) | 0 (0) |
| VELOUR | 113 | 59 (52.2) | 21 (18.5) | 13 (11.5) | 17 (15.0) | 3 (2.6) |
| RAISE | 136 | 119 (46.1) | 56 (21.7) | 18 (6.9) | 47 (18.2) | 18 (6.9) |

| **Supplementary Table 3. Adjusted variables of individual studies for multivariate models** | |
| --- | --- |
| Study | Adjusted covariates |
| Kitazume 2022 | Concomitant use of PPI (yes vs. no), age (10- year intervals), sex (male vs. female), primary site [right-sided colon vs. others (left-sided colon and/or rectal)], cancer stage [III high-risk (T4, N2, or both cancers) vs. III low-risk (T1, T2, or T3 and N1 cancers) vs. II], chemotherapy regimen (CapeOX vs. capecitabine), and RDI (10% intervals) |
| Wong 2019 | N/A |
| Sun 2016 | Age (> 68 years), gender, stage III, and poorer ECOG PS (ECOG >/=2) |
| Wang 2017 | N/A |
| AXEPT | Country, performance status, number of metastatic sites, previous use of oxaliplatin treatment, and concurrent bevacizumab treatment |
| HORIZON III | Age, gender, race, ECOG-PS, and serum CEA and lactate dehydrogenase (LDH) levels |
| N016966 | Age, gender, race, ECOG-PS, and serum CEA and lactate dehydrogenase (LDH) levels |
| AVF2107 | Age, gender, race, ECOG-PS, and serum CEA and lactate dehydrogenase (LDH) levels |
| Carrato 2013 | Age, gender, race, ECOG-PS, and serum CEA and lactate dehydrogenase (LDH) levels |
| VELOUR | Age, gender, race, ECOG-PS, and serum CEA and lactate dehydrogenase (LDH) levels |
| RAISE | Age, gender, race, ECOG-PS, and serum CEA and lactate dehydrogenase (LDH) levels |

| **Supplementary Table 4. Non-CRC studies assessing the association between PPI and capecitabine** | | | | | | | | |
| --- | --- | --- | --- | --- | --- | --- | --- | --- |
| Study | Sample size | PPI users,  n (%) | Study type | Cancer type | Chemotherapy regimen | Definition of PPI users | OS, HR (95% CI) | PFS, HR (95% CI) |
| Chu 2017^1^ | 545 | 229 (42%) | Post-hoc analysis of TRIO-013 trial | HER-2 (+) Gastroesophageal cancer | CapeOx +/- lapatinib | ≥ 20% overlap between PPI and treatment duration | 1.34 (1.06 to 1.62) | 1.55 (1.29 to 1.81) |
| Yang 2017^2^ | 891 | 252 (28%) | Post-hoc analysis of phase III RCT | M0/M1 Breast cancer  M0/M1 Gastric cancer | Capecitabine | During Rx | N/A | 1.01 (0.73 to 1.36)  1.13 (0.84 to 1.50) |
| Roberto 2020^3^ | 67 | 32 (48%) | Randomized controlled trial | GI tract cancers | Capecitabine | N/A | 0.89 (0.54 to 1.48) | 1.22 (0.75 to 2.00) |

**Reference:**

1. Chu MP, Hecht JR, Slamon D, et al. Association of Proton Pump Inhibitors and Capecitabine Efficacy in Advanced Gastroesophageal Cancer: Secondary Analysis of the TRIO-013/LOGiC Randomized Clinical Trial. *JAMA Oncol.* 2017;3(6):767-773.

2. Yang JY SH, Sandler RS, Stürmer T, Funk MJ, Lund JL. Does proton-pump inhibitor use diminish capecitabine efficacy in advanced cancer patients? . *Pharmacoepidemiology Drug Safety.* 2017;26:222-223.

3. Roberto M, Romiti A, Mazzuca F, et al. Combination Therapy of High-Dose Rabeprazole Plus Metronomic Capecitabine in Advanced Gastro-Intestinal Cancer: A Randomized Phase II Trial. *Cancers (Basel).* 2020;12(11).
